# Bridging Temporal and Cepstral Hemodynamic Intelligence: An Adaptive Multi-Domain Feature Fusion Framework for Multi-Class fNIRS Brain–Computer Interfaces

**DOI:** 10.3390/s26206427

**Published:** 2026-10-11

**Authors:** Muhammad Saad Bin Abdul Ghaffar, Hüseyin Metin Ertunç, Kaplan Kaplan, Kerem Kucuk, Khalid Yahya, Sajjad Ahmad Khan

**Affiliations:** 1 Department of Mechatronics Engineering, Kocaeli University, Kocaeli 41001, Türkiye; 2 Department of Software Engineering, Kocaeli University, Kocaeli 41001, Türkiye; 3 KOU Teknopark, Algorasoft Information Technologies Industry and Trade Co., Ltd., Kocaeli 41275, Türkiye; 4 Department of Electrical and Electronics Engineering, Faculty of Engineering and Architecture, Istanbul Gelisim University, Istanbul 34310, Türkiye

**Keywords:** brain-computer interface (BCI), feature fusion, adaptive multi-domain feature fusion (AMFF), functional near-infrared spectroscopy (fNIRS), mel-frequency cepstral coefficients (MFCC), multilayer perceptron (MLP), pattern recognition

## Abstract

Reliable four-class decoding remains challenging for functional near-infrared spectroscopy (fNIRS) brain–computer interfaces (BCIs), because a single feature domain captures only part of the hemodynamic response. This study proposes an Adaptive Multi-Domain Feature Fusion (AMFF) framework that combines six temporal hemodynamic descriptors with ten fNIRS-adapted Mel-frequency cepstral coefficients (MFCCs) and learns sample-dependent weights for the two domains before classification with a multilayer perceptron. The framework was evaluated on an open-access dataset of 29 participants performing left- and right-hand motor imagery, mental arithmetic, and rest, using subject-independent leave-one-subject-out cross-validation. AMFF achieves a mean accuracy of 95.6%, with precision, recall, and F1-score of 94.6%, 95.9%, and 95.3%, respectively, outperforming temporal only (90.7%), cepstral-only (95.0%), and direct concatenation (95.2%) representations, with a modest but consistent gain over direct concatenation across participants. Friedman and Holm-corrected Wilcoxon signed-rank tests confirmed significant differences for all four metrics; AMFF exceeded static concatenation for every participant, although by a small margin (0.2–0.7 percentage points). Most of the gain over temporal descriptors therefore comes from the cepstral domain, with adaptive weighting adding a small but consistent refinement. MFCC parameter-sensitivity, coefficient-ablation, and descriptor-ablation analyses further characterize the contribution of each domain.

## 1. Introduction

Brain–Computer Interfaces (BCIs) are based on the principle that neuronal activity within the brain cells can generate measurable physiological responses that encode physical actions or cognitive intentions [1]. These responses can be decoded and translated into control commands for external devices, enabling direct communication pathways between the brain and machines. This closed-loop interaction paradigm is formally described as the BCI cycle, encompassing signal acquisition, decoding, feedback, and adaptation mechanisms [2]. BCI technologies have demonstrated substantial potential in assisting individuals with impaired motor functions to interact with assistive devices, such as wheelchairs, neuroprostheses, and rehabilitation systems, using neural activity rather than residual muscular control [3,4]. Improving the neural decoding accuracy of brain signals remains a central challenge for advancing the reliability and practical utility of BCI systems [5]. Enhancements in decoding performance can reduce user dependency, training time, and cognitive load, thereby supporting more effective BCI-based communication, assistance, and rehabilitation [6].

BCI systems are commonly categorized into invasive and non-invasive approaches. Invasive BCIs rely on surgically implanted electrodes that provide high-fidelity neural recordings but are associated with substantial surgical risks, long-term stability concerns, and ethical limitations. In contrast, non-invasive BCIs acquire neural information without surgical intervention, making them more suitable for widespread adoption and long-term use. Consequently, non-invasive modalities dominate current BCI research and clinical translation efforts [6,7]. Several non-invasive neuroimaging techniques have been explored for BCI development, including functional magnetic resonance imaging (fMRI), electroencephalography (EEG), and functional near-infrared spectroscopy (fNIRS). Although fMRI provides excellent spatial resolution, its high cost, lack of portability, and delayed hemodynamic response limit its applicability for real-time BCI systems. EEG offers superior temporal resolution but suffers from poor spatial specificity and susceptibility to motion artifacts and electromagnetic noise. fNIRS has therefore emerged as a promising alternative because of its favorable balance between spatial and temporal characteristics, immunity to electromagnetic interference, and robustness against moderate motion artifacts. These properties make fNIRS particularly suitable for wearable and portable BCI applications [8,9].

fNIRS measures task-related hemodynamic responses by quantifying changes in oxygenated hemoglobin (HbO) and deoxygenated hemoglobin (HbR) concentrations using near-infrared light in the wavelength range of approximately 600–1000 nm. These concentration variations, denoted as $\Delta c_{\mathrm{HbO}}\left(t\right)$ and $\Delta c_{\mathrm{HbR}}\left(t\right)$, are computed using the modified Beer-Lambert law [10]. The fundamental principle of near-infrared spectroscopy was introduced by Jobsis in 1977, and an optimal source-detector separation of approximately 3–4 cm is generally employed to provide adequate cortical sensitivity while reducing contamination from extracerebral tissues [11]. Owing to these characteristics, fNIRS has been widely adopted in cognitive neuroscience, motor imagery studies, neurorehabilitation, and BCI research.

**Figure 1:**
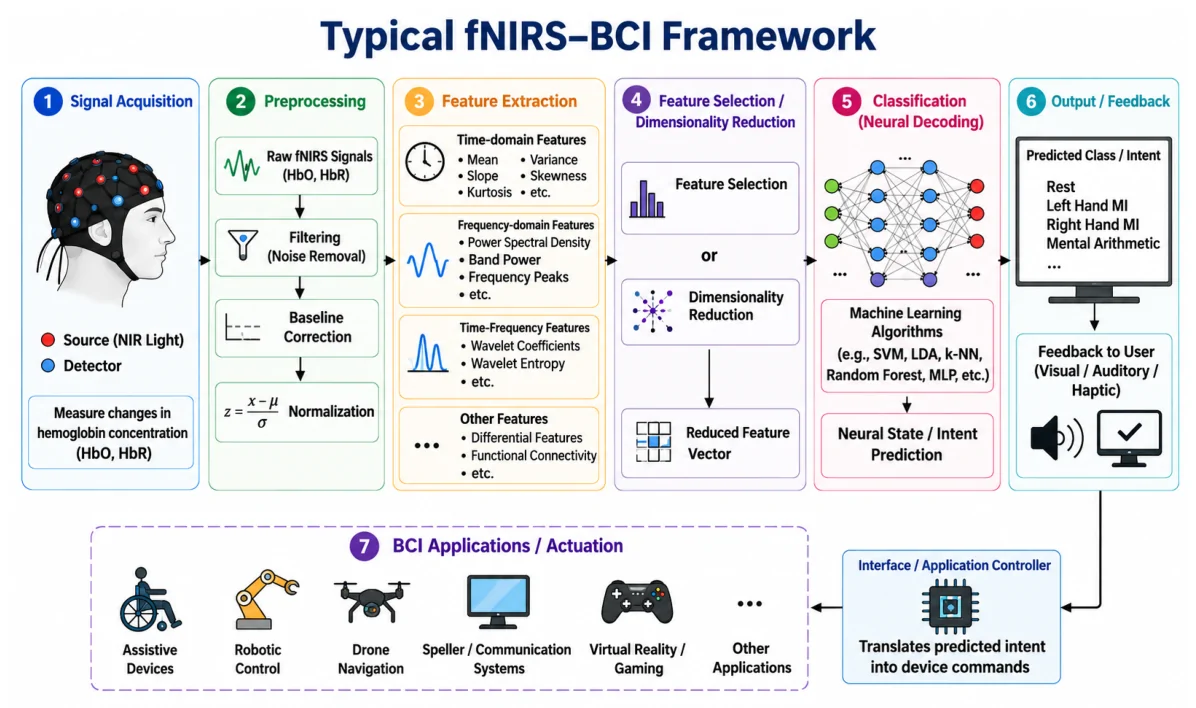
Schematic representation of the fNIRS-BCI framework, illustrating the multi-stage pipeline from signal acquisition to application control.

The architecture of a typical fNIRS-BCI framework, illustrated in Figure 1, follows a structured multi-stage pipeline encompassing signal acquisition, preprocessing, feature extraction, neural decoding, and application control. Initially, neural activity is acquired through the selected neuroimaging modality. The recorded signals are subsequently processed to mitigate physiological noise, motion artifacts, and instrumental interference [12]. After preprocessing, discriminative features are extracted to characterize the underlying neural states and are subsequently provided to a classifier for decoding distinct control commands. Finally, an application controller interprets these commands to operate external devices or software interfaces [7]. During high-performance BCI operation, real-time neurofeedback may also be integrated to facilitate user adaptation and volitional self-regulation of neural activity, thereby establishing a closed-loop system [2]. This modular architecture provides the foundation for BCI systems used in communication, assistive technologies, and neurorehabilitation [6].

Despite substantial progress in fNIRS-based BCIs, achieving accurate and robust neural decoding, particularly for multi-class paradigms, remains challenging. fNIRS signals exhibit a relatively low signal-to-noise ratio (SNR), pronounced inter-subject variability, and intrinsically delayed and slowly varying hemodynamic responses. These characteristics can limit the reliability of neural-state discrimination and make it difficult for a single feature representation to capture all task-relevant information contained in the hemodynamic response. Consequently, recent fNIRS research has increasingly moved beyond isolated feature sets toward richer representation learning and information integration, including multi-domain and multimodal feature fusion [12,13,14,15]. Recent reviews of fNIRS-based artificial intelligence and deep learning have also reported increasing use of convolutional, recurrent, graph-based, and Transformer architectures for learning discriminative representations directly from fNIRS signals [15,16,17,18]. However, the effectiveness of neural decoding remains strongly dependent on how task-relevant information is represented and integrated before classification.

Feature representation is therefore a critical component of fNIRS-BCI systems. Conventional approaches have predominantly relied on handcrafted temporal and statistical descriptors, including mean, variance, standard deviation, slope, skewness, kurtosis, and peak-related characteristics. These descriptors provide compact and interpretable representations of the amplitude, variability, distribution, and morphological characteristics of the hemodynamic response and have been successfully applied to motor imagery, mental workload, and other cognitive-state classification problems [19,20,21]. However, temporal descriptors characterize the signal primarily from a temporal-statistical perspective and do not explicitly represent its spectral structure. Deep learning approaches can partially address this limitation by learning representations from raw or transformed fNIRS signals. For example, Ma et al. demonstrated the effectiveness of convolutional neural networks for fNIRS motor imagery classification, while subsequent studies investigated convolutional, recurrent, graph-based, and attention-based architectures [22,23,24,25]. Although these approaches demonstrate the potential of richer representation learning, they do not necessarily exploit explicitly engineered complementary feature domains.

The increasing recognition of complementary temporal and spectral information has motivated the incorporation of frequency-domain and time-frequency representations into fNIRS analysis. The physiological information contained in fNIRS is not restricted to signal amplitude in the time domain; task-related hemodynamic responses also exhibit characteristic low-frequency spectral variations. This has motivated the use of frequency-domain transformations, spectrograms, time-frequency representations, and frequency-enhanced neural architectures. Wang et al. investigated the implications of delayed hemodynamic responses for fNIRS classification, emphasizing the importance of appropriately representing the temporal characteristics of the hemodynamic response [26]. Khani et al. proposed a two-dimensional discrete orthonormal Stockwell transform (2D-DOST) based time-frequency representation and fused deep features extracted from multiple representations of HbO, HbR, and their linear combinations. Their results demonstrated that joint fusion of multiple time-frequency representations can improve classification compared with relying on a single representation [14]. Li et al. introduced FFTNet, a frequency-enhanced patch network designed to explicitly exploit frequency information in fNIRS signals for driving-fatigue detection [27]. All of the above studies indicate that frequency characteristics provide useful complementary information that can be considered alongside conventional temporal representations.

Among the available spectral representations, MFCCs offer several characteristics that make them particularly suitable as a complementary feature domain for the present fNIRS framework. Power spectral density (PSD) features directly quantify the distribution of signal power across frequency and have been widely used in neural-signal analysis; however, PSD-based representations generally retain the frequency-bin or band-power structure of the spectrum and therefore may require additional band selection or dimensionality reduction when used as handcrafted feature vectors [28,29]. Wavelet representations provide a valuable alternative because they characterize signal variations jointly in time and frequency and have been widely applied to fNIRS analysis. However, wavelet-based analysis requires choices regarding the wavelet family, scaling/decomposition levels, and the coefficients or frequency bands retained for subsequent analysis. These choices can affect the resulting representation, while deeper decompositions may introduce redundant features and increase computational cost [30,31]. In contrast, MFCC extraction first aggregates spectral energy through a filter bank, applies logarithmic compression, and subsequently uses the discrete cosine transform (DCT) to obtain a compact set of approximately decorrelated cepstral coefficients [32,33]. This processing provides a low-dimensional summary of the overall spectral envelope rather than retaining individual spectral bins, making MFCCs attractive when a compact frequency-domain representation is required to complement temporal statistical descriptors. The choice of MFCCs in this work is therefore not based on an assumption that they universally outperform PSD or wavelet features; rather, MFCCs were selected because their compact cepstral representation provides a distinct and computationally manageable description of spectral characteristics that can be adaptively combined with temporal hemodynamic descriptors. For the very low-frequency range of fNIRS, the Mel-frequency warping itself is close to linear; consequently, the value of the adopted MFCC representation is attributed primarily to the combination of spectral filter-bank aggregation, logarithmic compression, and DCT-based dimensionality reduction rather than to perceptual frequency warping [33].

Although the perceptual motivation of the Mel scale is specific to auditory processing and should not be interpreted as a physiological model of cerebral hemodynamics, MFCCs have already demonstrated feasibility for fNIRS-BCI classification: Ghaffar et al. applied MFCCs to four-class fNIRS-BCI decoding and reported that the cepstral representation provided discriminative information beyond conventional statistical features [34]. In the present work, MFCCs are therefore employed not as a replacement for PSD or wavelet representations, but as a compact, complementary cepstral description of the hemodynamic response.

Feature fusion has also been extensively investigated in hybrid EEG-fNIRS BCIs because the two modalities provide complementary physiological information. EEG offers high temporal resolution, whereas fNIRS provides comparatively richer spatial information and is less susceptible to electrical interference. Their combination can therefore provide information that may not be available from either modality independently. Deligani et al. proposed a mutual-information-based hybrid classification framework for EEG-fNIRS fusion, demonstrating the potential of exploiting complementary information from the two modalities [35]. He et al. developed a multimodal multitask neural network for EEG-fNIRS motor imagery classification and demonstrated the benefits of jointly learning representations from both modalities [36]. Bunterngchit et al. subsequently combined selective channel representations and spectrogram-based representations for simultaneous EEG-fNIRS classification [37]. These studies establish feature and multimodal fusion as important strategies for exploiting complementary neural information.

The fusion strategy itself has progressively evolved from conventional feature concatenation toward more sophisticated decision-level, attention-based, correlation-aware, and multi-granularity mechanisms. Classical feature-level fusion generally concatenates feature vectors extracted independently from different modalities or representations before classification. Although straightforward, concatenation does not explicitly account for differences in the discriminative importance of the contributing feature groups. Decision-level fusion provides an alternative in which individual classifiers generate prediction scores that are subsequently combined. Rabbani and Islam investigated deep-learning-based decision fusion for EEG-fNIRS classification, demonstrating the potential of combining modality-specific decisions rather than directly concatenating their features [38]. Seno et al. incorporated evidence theory into EEG-fNIRS fusion to model uncertainty in individual modality decisions before performing decision-level integration [39]. These studies indicate that the fusion stage can substantially influence final classification performance and that simple combination strategies may not fully exploit complementary information.

Deep learning has further enabled fusion mechanisms in which the relative contribution of individual modalities or feature representations can be learned automatically. Qin et al. proposed ECA-FusionNet for hybrid EEG-fNIRS motor imagery classification, incorporating feature-level and decision-level fusion with attention mechanisms [40]. Li et al. introduced cross-modality attentional fusion for EEG-fNIRS cognitive-state decoding, while Shi et al. developed a spatial–temporal convolution and dual-attention framework for multimodal EEG-fNIRS fusion [41,42]. Zhang et al. proposed TSFNet to model temporal and spatial information within a hybrid BCI framework [43]. More recently, Yuan et al. introduced IIMCNet to model intra- and inter-modality correlations in hybrid EEG-fNIRS data [44], while Zhang et al. proposed MGFNet, which employs multi-granularity fusion with coupling-guided sparse routing for hybrid EEG-fNIRS decoding [45]. Xu et al. introduced a disentangled multimodal spatiotemporal learning framework that separates common and modality-specific representations while retaining cross-modal information [46]. Wang et al. further proposed a decoupled bidirectional spatiotemporal fusion network incorporating cross-modal guidance, adaptive temporal alignment, and symmetric cross-attention fusion [47]. All of the above developments demonstrate a broader transition from static feature concatenation toward learned feature interaction, adaptive weighting, alignment, and selective information integration.

Most recent feature-fusion research in fNIRS-based BCI systems continues to focus on multimodal fusion, particularly the integration of EEG and fNIRS, rather than the adaptive fusion of heterogeneous feature domains derived from a single fNIRS modality. Multimodal fusion introduces additional sources of heterogeneity, including differences in sampling rate, temporal resolution, spatial organization, and physiological origin. In contrast, feature-domain fusion within fNIRS can exploit complementary descriptions of the same underlying hemodynamic response without introducing an additional sensing modality. Recent systematic reviews similarly identify multimodal integration, representation learning, and adaptive fusion as important directions in the continued development of fNIRS-based deep learning systems [15,18].

Despite these advances, feature-domain fusion within single-modality fNIRS remains substantially less explored than multimodal EEG-fNIRS fusion. An important example is the study by Qiu et al. [13], who explicitly evaluated multi-domain fNIRS representations by combining temporal statistical descriptors with power spectral density (PSD) features from HbO and HbR. Their results demonstrated that combining temporal and frequency-domain features improved classification compared with individual feature domains, confirming that complementary information can be exploited within a single fNIRS modality. However, their framework primarily used conventional PSD representations and employed feature selection and progressive learning for subsequent fusion rather than learning the relative contribution of heterogeneous feature domains themselves. Similarly, Khani et al. [14] investigated fusion of multiple time-frequency representations derived from fNIRS signals, including HbO, HbR, and their combinations, demonstrating the value of integrating complementary representations within the same modality. Wen et al. (2025) proposed VisiTempNet, fusing Gramian angular difference field image features with time-series features through learnable weights, reaching 76.65% accuracy on the fNIRS2MW dataset [48]. Dale et al. (2023) demonstrated spatial–temporal 3D-CNN feature extraction on high-density frequency-domain fNIRS topographical maps, improving subject-independent motor-task classification over a temporal CNN [49]. Akhter et al. (2025) stacked deep features from CNN, LSTM, and Bi-LSTM with FFT enhancement for hand-gripping fNIRS-BCI, reaching 90.11% versus 79% to 85% for conventional networks [50]. Lyu et al. (2025) combined multi-scale fused features with an adaptively gated improved Transformer, reaching up to 77.97% accuracy in task classification, with ablations confirming fused features beat single-feature inputs [51]. These studies establish the feasibility and potential of single-modality fNIRS multi-domain fusion, but they leave open the question of how substantially different handcrafted feature domains, particularly temporal statistical and cepstral representations, can be integrated and weighted adaptively.

Within single-modality fNIRS analysis, existing studies have demonstrated the usefulness of temporal, spectral, and time-frequency representations individually or through specific fusion schemes. PSD-based representations emphasize the distribution of signal power across frequency, whereas wavelet-based methods provide localized time-frequency information. Cepstral representations offer a different form of spectral description by compressing the spectral envelope into a compact coefficient space. Despite the availability of these complementary representations, systematic investigation of the interaction between conventional temporal hemodynamic descriptors and MFCC-based cepstral descriptors within a single fNIRS modality remains comparatively limited. In particular, static concatenation of heterogeneous feature vectors does not account for the possibility that the relative discriminative value of each feature domain may vary across subjects and cognitive states. An effective fusion mechanism should therefore not only combine different feature spaces but also determine their relative contributions during model optimization. This gap motivates the development of an adaptive multi-domain feature fusion strategy that integrates temporal hemodynamic descriptors with fNIRS-adapted MFCCs.

Building on this research direction, this study proposes an Adaptive Multi-Domain Feature Fusion (AMFF) framework for four-class fNIRS-based BCI neural decoding. The proposed framework extracts two complementary representations from the same preprocessed hemodynamic signals: six temporal hemodynamic descriptors and ten fNIRS-adapted Mel-Frequency Cepstral Coefficients (MFCCs). Instead of assigning equal importance to the two feature domains, AMFF employs trainable weighting mechanisms to estimate their relative contributions and subsequently integrates the weighted representations into a unified feature space before classification. The resulting representation is designed to jointly capture temporal hemodynamic morphology and cepstral characteristics, thereby providing a more comprehensive description of the neural response for four-class fNIRS-BCI decoding.

Table 1 summarizes representative feature-fusion approaches reported in fNIRS and hybrid EEG-fNIRS BCI systems, highlighting the evolution from conventional feature and decision-level fusion toward attention-based and adaptive representation learning.

The main contributions of this study are summarized as follows:

1. Development of an Adaptive Multi-Domain Feature Fusion (AMFF) framework that adaptively integrates complementary temporal hemodynamic and MFCC-based cepstral representations for four-class fNIRS-BCI neural decoding.
2. Investigation of MFCC-based cepstral descriptors as a complementary frequency-domain representation to conventional temporal hemodynamic descriptors within a single-modality fNIRS framework.
3. Comparison of temporal descriptors, MFCC-based cepstral descriptors, static concatenation, and AMFF using the same MLP decoder under subject-independent leave-one-subject-out validation across 29 participants, separating the benefit of combining the two domains from that of adaptive weighting.
4. Analysis of the learned adaptive weights, an MFCC parameter-sensitivity study, and ablations of both the temporal descriptors and the individual MFCC coefficients.
5. Statistical assessment (Friedman and Holm-corrected Wilcoxon tests with effect sizes) of accuracy, precision, recall, and F1-score.

Under the subject-independent leave-one-subject-out (LOSO) evaluation used in this study, the proposed AMFF framework achieved a mean four-class fNIRS-BCI decoding accuracy of 95.6%. This result compares favorably with the performance reported in several existing fNIRS-BCI studies. Because published fNIRS-BCI studies differ in datasets, numbers and types of task classes, preprocessing pipelines, feature representations, classifiers, and validation protocols, these comparisons should be regarded as contextual rather than as a direct ranking of methods. By adaptively integrating complementary temporal and cepstral feature representations, the proposed approach provides a robust and scalable framework for high-accuracy neural decoding.

The remainder of this paper is organized as follows. Section 2 details the experimental methodology, encompassing participant demographics, the experimental protocol, and signal acquisition. This section further describes the preprocessing procedures, extraction of cepstral and temporal hemodynamic descriptors, and the Adaptive Multi-Domain Feature Fusion Framework (AMFF) with the MLP-based neural decoder. Section 3 presents the experimental results and performance analysis, while Section 4 provides a comparative discussion against existing benchmarks and contemporary studies. Finally, Section 5 summarizes the conclusions and outlines future research directions.

## 2. Materials and Methods

**Figure 2:**
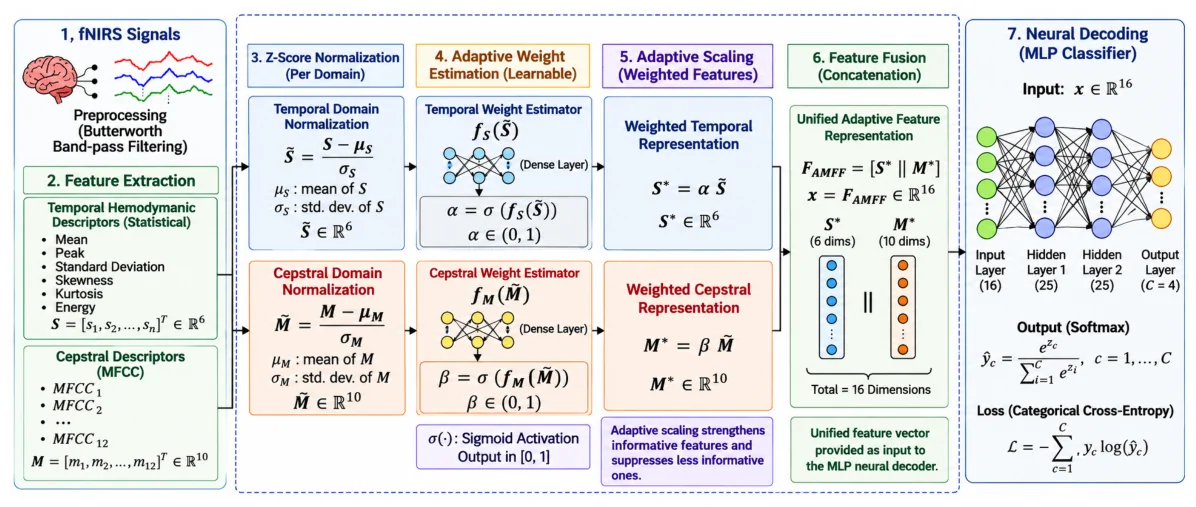
Illustration of the methodological framework.

This section details the comprehensive methodological framework developed for this study. Figure 2 provides an overview of the complete methodological workflow adopted in this study.

### 2.1. Experimental Protocols

This study utilized the publicly available open-access fNIRS dataset introduced by Shin et al. [52]. The dataset was acquired using a multi-channel continuous-wave fNIRS system (NIRScout, NIRx GmbH, Berlin, Germany) and includes recordings of motor imagery (MI), comprising left-hand motor imagery (LHMI) and right-hand motor imagery (RHMI), mental arithmetic (MA), and resting-state conditions. Data were collected from 29 healthy adult participants (14 males and 15 females, mean age 28.5±3.7 years, age range 17–34 years) using dual wavelengths of 760 nm and 850 nm, which are commonly employed for quantifying changes in oxygenated (HbO) and deoxygenated hemoglobin (HbR).

The fNIRS signals were sampled at a frequency of 12.5 Hz. Data acquisition targeted cortical regions relevant to the experimental tasks, including the prefrontal cortex (PFC) for mental arithmetic and the primary motor cortex (M1) for motor imagery. In addition, channels covering the visual cortex were included, as visual stimulation was required for task instruction and cue presentation. This multi-region configuration enables comprehensive capture of task-related hemodynamic responses while accounting for visually evoked activity.

The selected cortical regions are consistent with established neurophysiological evidence indicating that the primary motor cortex is optimally suited for decoding motor imagery tasks, while the prefrontal cortex plays a dominant role in mental arithmetic and executive cognitive processing. The inclusion of both regions follows standard practice in fNIRS-BCI research and supports reliable task discrimination across cognitive and motor paradigms [53,54].

The diverse cognitive and motor paradigms included in this dataset provide a suitable benchmark for evaluating the effectiveness of the proposed adaptive multi-domain feature fusion framework under a challenging four-class neural decoding scenario.

### 2.2. Experimental Paradigm

The experimental paradigm followed the protocol described in the publicly available dataset of Shin et al. [52]. Participants were seated comfortably in front of a display throughout the experiment and were instructed to minimize unnecessary head and body movements to reduce motion-related artifacts in the fNIRS recordings. Visual instructions were presented before each trial to ensure consistent task execution.

To ensure that genuine motor imagery (MI), rather than visually driven imagery, was performed, participants were explicitly instructed to engage in kinesthetic motor imagery, imagining repeatedly opening and closing their hands as if grasping a ball. During the MI experiment, all participants were inexperienced with MI tasks, thereby avoiding performance bias related to prior motor imagery training. For MI trials, a black arrow with a left- or right-pointing arrowhead was displayed at the center of the screen for 2 s, serving as a visual cue for left hand motor imagery (LHMI) or right hand motor imagery (RHMI), respectively.

For mental arithmetic (MA) tasks, a subtraction instruction consisting of a three-digit number minus a one-digit or two-digit number (e.g., 223−23) was presented at the center of the screen for 2 s. During this instruction phase, participants were asked to memorize the numbers. Once the visual instruction disappeared, they continuously performed the subtraction mentally until the end of the task period.

The experiment consisted of four task conditions: right hand motor imagery (RHMI), left hand motor imagery (LHMI), mental arithmetic (MA), and resting state (Rest). For each participant, the experiment comprised three sessions, each preceded and followed by a 60 s resting period. Each session comprised 20 trials, with each trial beginning with a 2 s visual instruction, followed by a 10 s task execution period, and concluding with a 15–17 s inter-trial rest interval.

Although the dataset contains 3480 trial samples in total across all 29 participants (120 trials per participant, corresponding to 30 trials for each of the four experimental classes), the trial count is not interpreted as the number of statistically independent experimental units. Instead, participant identity was used as the unit of data partitioning and evaluation, thereby ensuring that all trials from a given participant remained within the same fold.

**Figure 3:**
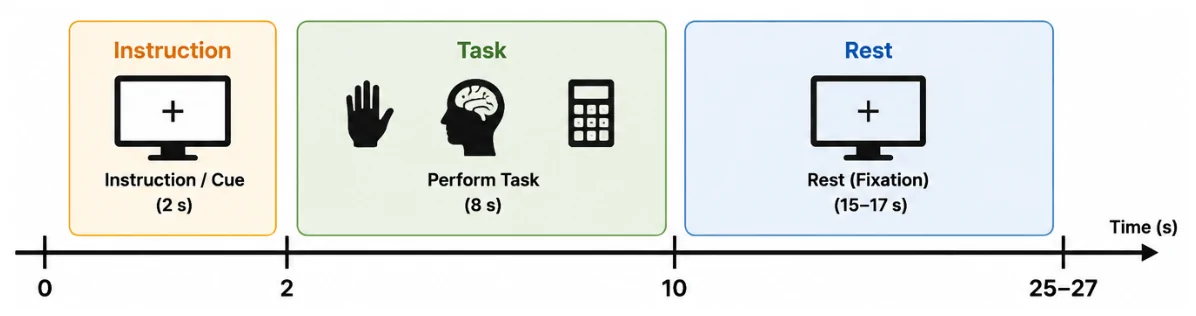
Experimental paradigm showing the sequence and duration of the instruction, task-performance, and resting periods.

The relatively long task duration was intentionally selected to collect sufficient hemodynamic data for reliable temporal hemodynamic descriptor extraction. A larger number of observations improves the robustness of both temporal hemodynamic descriptors and MFCC-based cepstral feature extraction while providing sufficient information for stable classifier training. Since learning complementary temporal and cepstral feature representations and their adaptive fusion constitutes a primary objective of this study, sufficient high-quality data were required for reliable feature extraction and subsequent neural decoding. A schematic overview of the experimental paradigm is illustrated in Figure 3 [52].

### 2.3. Signal Acquisition

**Figure 4:**
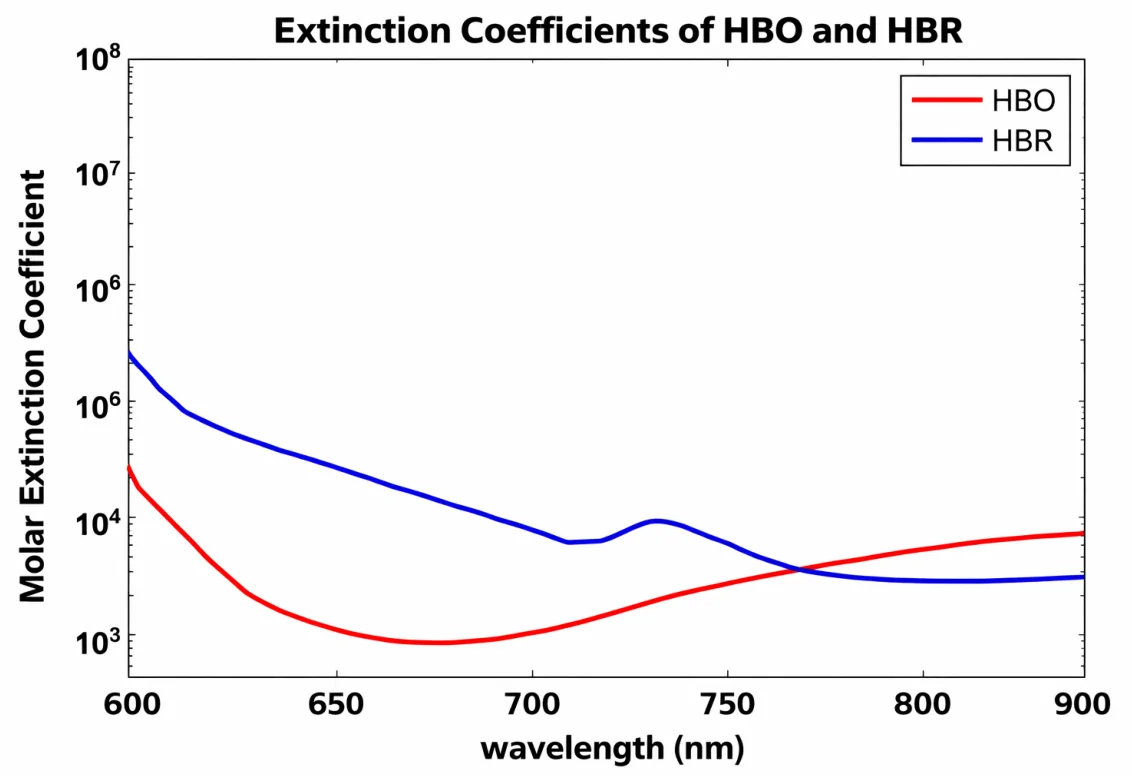
Wavelength-dependent molar extinction coefficients of oxygenated hemoglobin (HbO) and deoxygenated hemoglobin (HbR) in the near-infrared range (590–900 nm). The curves illustrate the distinct wavelength-dependent absorption characteristics of HbO and HbR.

Functional near-infrared spectroscopy (fNIRS) quantifies task-related changes in oxygenated hemoglobin (HbO) and deoxygenated hemoglobin (HbR) concentrations by transmitting near-infrared light through the scalp and skull. In this study, light sources operating at wavelengths of 760 nm and 850 nm were employed, as these wavelengths exhibit distinct absorption characteristics for HbO and HbR, enabling their reliable separation. Figure 4 illustrates the difference in extinction coefficients of HbO and HbR across the near-infrared spectrum.

Both oxyhemoglobin and deoxyhemoglobin selectively absorb near-infrared light, while unabsorbed photons propagate through biological tissue and are either scattered or detected at the scalp surface. The fNIRS detectors measure the intensity of the returning photons, which reflects changes in optical density induced by variations in cerebral blood oxygenation. To estimate the underlying hemodynamic responses, the measured optical density changes were converted into relative HbO and HbR concentration changes using the Modified Beer–Lambert Law (MBLL), which accounts for wavelength-dependent absorption, differential pathlength factors, and scattering effects in biological tissue. The MBLL provides a linear relationship between changes in optical density and corresponding hemoglobin concentration variations and is widely adopted in fNIRS signal processing [10].

(1) $$\left[\begin{array}{c} \Delta c_{\mathrm{HbO}}\left(t\right)\\ \Delta c_{\mathrm{HbR}}\left(t\right) \end{array}\right]=\frac{{\left(\left[\begin{array}{cc} \varepsilon_{\mathrm{HbO}}\left(\lambda_{1}\right) & \varepsilon_{\mathrm{HbR}}\left(\lambda_{1}\right)\\ \varepsilon_{\mathrm{HbO}}\left(\lambda_{2}\right) & \varepsilon_{\mathrm{HbR}}\left(\lambda_{2}\right) \end{array}\right]\right)}^{-1}\left[\begin{array}{c} \Delta A(t,\lambda_{1})\\ \Delta A(t,\lambda_{2}) \end{array}\right]}{l\times d}$$

where *l* is the distance separating the emitter and the receiver, *d* is the differential pathlength factor (DPF), which accounts for the increase in photon path length due to scattering in biological tissue, and $\Delta A(t,\lambda_{1})$ and $\Delta A(t,\lambda_{2})$ denote the changes in optical density measured at time *t* for light of wavelengths $\lambda_{1}$ and $\lambda_{2}$, respectively. The terms $\varepsilon_{\mathrm{HbO}}\left(\lambda_{1}\right)$ and $\varepsilon_{\mathrm{HbR}}\left(\lambda_{1}\right)$ represent the wavelength-dependent molar extinction coefficients of oxyhemoglobin (HbO) and deoxyhemoglobin (HbR) at wavelength $\lambda_{1}$, while $\varepsilon_{\mathrm{HbO}}\left(\lambda_{2}\right)$ and $\varepsilon_{\mathrm{HbR}}\left(\lambda_{2}\right)$ represent the corresponding coefficients at wavelength $\lambda_{2}$. These coefficients are tabulated constants of the absorbing species and are therefore written without a Δ operator. Finally, $\Delta c_{\mathrm{HbO}}\left(t\right)$ and $\Delta c_{\mathrm{HbR}}\left(t\right)$ denote the changes in the concentrations of HbO and HbR at time *t*. The resulting HbO and HbR concentration signals constitute the primary hemodynamic measurements used throughout the subsequent preprocessing, feature extraction, and neural decoding stages.

### 2.4. Signal Processing

To improve the quality of the hemodynamic signals prior to feature extraction, several preprocessing steps were applied to the acquired HbO and HbR concentration signals. Butterworth filtering was selected to process the oxygenated and deoxygenated hemoglobin concentration signals, denoted as $c_{\mathrm{HbO}}\left(t\right)$ and $c_{\mathrm{HbR}}\left(t\right)$, respectively because of its maximally flat passband response, which effectively suppresses physiological and instrumental noise while preserving the slowly varying hemodynamic responses characteristic of fNIRS signals. Both low-frequency drift and high-frequency instrumental noise were attenuated, along with physiological artifacts arising from respiration (0.3–0.5 Hz), blood pressure oscillations (Mayer waves), and cardiac activity (1–1.5 Hz), by employing a sixth-order zero-phase bandpass filter with a passband of 0.01–0.3 Hz [55,56,57,58,59]. Following bandpass filtering, the signals underwent mean removal and baseline correction to suppress residual offsets. Baseline correction was performed by subtracting the mean signal amplitude computed from the corresponding baseline period, thereby compensating for slow baseline shifts and inter-trial offsets. Finally, each channel was standardized using z-score normalization to ensure a consistent numerical scale across channels and participants prior to feature extraction.

Primary feature extraction was performed on the HbO signal due to its higher signal-to-noise ratio in task-evoked cerebral oxygenation responses. However, to rigorously evaluate chromophore-level contributions as requested during peer review, parallel feature extraction and classification were also conducted for HbR-only and combined HbO+HbR representations using the identical preprocessing, feature extraction, AMFF fusion, and LOSO cross-validation pipeline. HbO was maintained as the baseline signal throughout the primary multi-domain experiments to ensure a controlled comparison between the temporal and cepstral feature representations.

### 2.5. Multi-Domain Hemodynamic Descriptors

Feature selection and feature engineering play a critical role in the performance of neural decoding systems. Features constitute the fundamental inputs upon which neural decoding models are built, and their relevance directly influences prediction accuracy, robustness, and generalizability. Well-designed feature representations enhance discriminative capability while reducing overfitting by suppressing redundant or noisy information. In contrast, irrelevant or highly correlated features can degrade model performance by increasing complexity and variance. Consequently, effective feature engineering and selection are essential for improving neural decoding accuracy and model interpretability in fNIRS based brain–computer interface (BCI) systems [54,60].

In fNIRS-BCI research, most existing studies have relied primarily on temporal hemodynamic descriptors (such as mean, variance, slope, skewness, and kurtosis) which describe amplitude and distributional properties of hemodynamic responses [53]. Although these descriptors effectively characterize the amplitude and statistical distribution of hemodynamic responses, they do not explicitly capture complementary spectral information that can subsequently be summarized through cepstral analysis.

In this study, features were extracted from two complementary domains. Since fNIRS signals are inherently recorded in the time domain, statistical features were first computed directly from the time-domain signals and are referred to as temporal descriptors. Subsequently, the same signals were transformed into a spectral representation as an intermediate processing step for MFCC computation. The resulting Mel-Frequency Cepstral Coefficients (MFCCs) constitute cepstral feature representations, which compactly encode the spectral envelope of the hemodynamic signals. MFCCs provide a compact cepstral representation of the spectral envelope while preserving discriminative information associated with the underlying hemodynamic dynamics, and have demonstrated strong performance in multi-class fNIRS-BCI neural decoding [34]. By integrating complementary temporal and MFCC-based cepstral descriptors through the proposed Adaptive Multi-Domain Feature Fusion Mechanism (AMFF), the framework aims to exploit complementary information across both domains to improve neural decoding robustness and accuracy.

Accordingly, the proposed framework constructs two complementary feature representations: (i) temporal hemodynamic descriptors extracted directly from the preprocessed signals and (ii) MFCC-based cepstral descriptors derived from the spectral envelope. These representations are subsequently adaptively fused through the proposed AMFF to form a unified multi-domain representation for neural decoding.

#### 2.5.1. Temporal Hemodynamic Descriptor

Six temporal hemodynamic descriptors, Signal Mean (SM), Standard Deviation (SD), Signal Variance (SV), Skewness (SK), Kurtosis (KR), and Signal Peak (SP), were computed from the preprocessed HbO signals for each of the four experimental tasks [3,20,53,54,61]. These descriptors are among the most widely used handcrafted temporal features in fNIRS-BCI research because they characterize complementary statistical properties of hemodynamic responses.

The signal mean (SM) was computed as

(2) $$\mathrm{SM}=\frac{1}{N}\sum_{i=1}^{N}x_{i},$$

where *N* denotes the total number of samples and $x_{i}=\Delta c_{\mathrm{HbO}}\left(t_{i}\right)$ represents the HbO concentration at the *i*-th sampling instant.

Skewness quantifies the asymmetry of the signal distribution with respect to its mean and is defined as

(3) $$\mathrm{Skew}=E\left[{\left(\frac{x_{i}-\mu }{\sigma }\right)}^{3}\right],$$

where E[·] denotes the expected value, μ is the mean of the signal, and σ is the standard deviation.

Kurtosis, which measures the peakedness of the signal distribution, was calculated as the fourth standardized moment (i.e., the raw, non-excess definition, for which a Gaussian distribution yields a value of 3),

(4) $$\mathrm{Kurt}=E\left[{\left(\frac{x_{i}-\mu }{\sigma }\right)}^{4}\right].$$

The standard deviation of the signal was computed as

(5) $$\sigma =\sqrt{\frac{\sum_{i=1}^{N}{\left(x_{i}-\mu \right)}^{2}}{N-1}},$$

where *N* is the total number of samples, μ is the mean value, and $x_{i}$ represents each individual sample. The unbiased (N−1) normalization is used consistently for both the standard deviation and the variance below, so that Equations (5) and (6) describe the same quantity.

Signal variance, which reflects the variability of the signal, was determined as

(6) $$\sigma^{2}=\frac{\sum_{i=1}^{N}{\left(x_{i}-\mu \right)}^{2}}{N-1}.$$

Here, *N* denotes the total number of samples, μ is the signal mean, and $x_{i}$ represents each signal sample.

The signal peak was obtained by identifying the maximum amplitude value of the signal over the task window.

**Figure 5:**
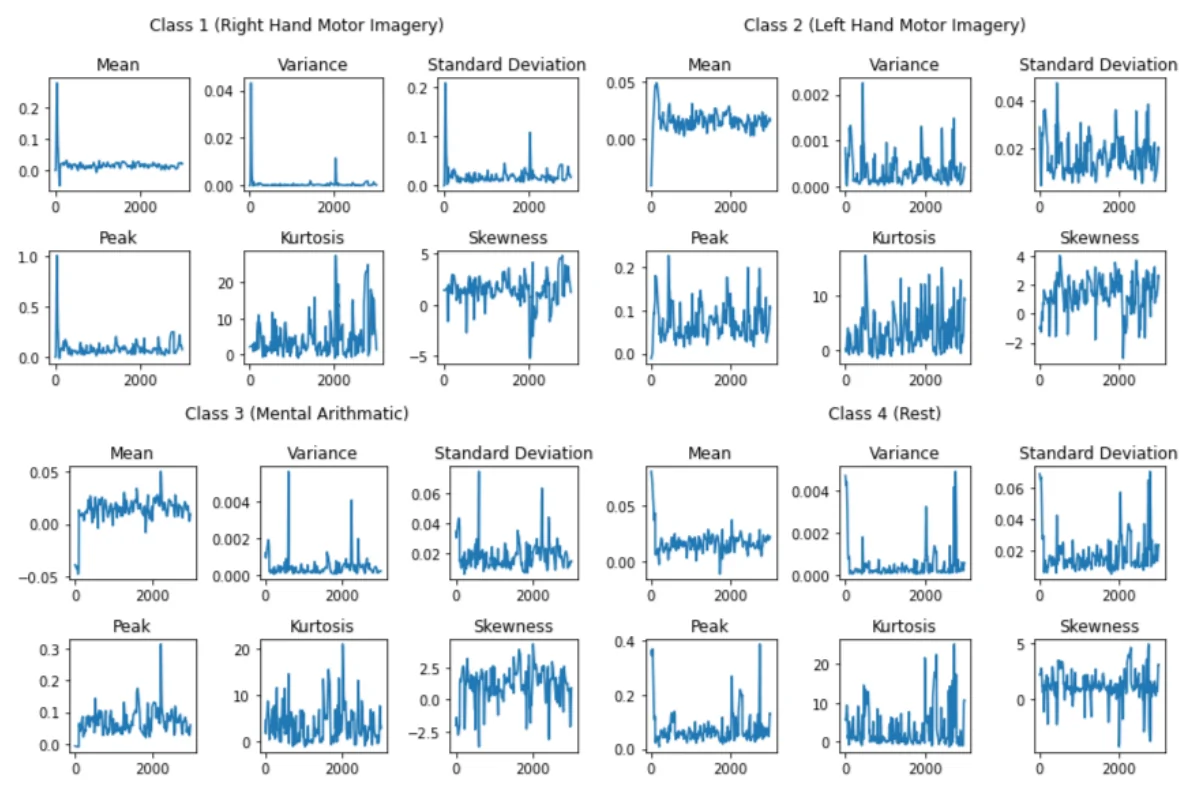
Temporal profiles of the extracted statistical descriptors (mean, variance, standard deviation, peak, kurtosis, and skewness) for the four motor-imagery classes of Subject 1. Y-axis scales are independently determined for each descriptor according to its value range and are therefore not directly comparable across different descriptor types.

Figure 5 illustrates all six temporal hemodynamic descriptors extracted from the data corresponding to the four experimental classes of Subject 1.

#### 2.5.2. Cepstral Hemodynamic Representation

Mel-frequency cepstral coefficients (MFCCs) are widely used in automatic speech recognition and speaker identification systems due to their ability to provide a compact cepstral representation of the spectral envelope. Since their introduction by Davis and Mermelstein in the early 1980s, MFCCs have remained a state-of-the-art feature extraction technique in audio signal processing and pattern recognition applications [33]. Beyond speech processing, cepstral and MFCC-based features have also been successfully applied to biomedical signal analysis, including electroencephalography (EEG)–based brain–computer interface (BCI) systems and neural activity neural decoding [34,62,63]. The Mel scale is obtained by applying a nonlinear, approximately logarithmic transformation to the linear frequency axis. In human auditory perception, equal perceptual differences in pitch correspond to equal distances on the Mel scale rather than equal linear frequency intervals. For example, the perceptual difference between 100 Hz and 200 Hz is more pronounced than that between 1000 Hz and 1100 Hz, despite identical linear frequency spacing. This perceptual nonlinearity motivated the design of the Mel scale, which effectively models human auditory sensitivity by emphasizing low-frequency components while compressing higher-frequency bands [33].

**Figure 6:**
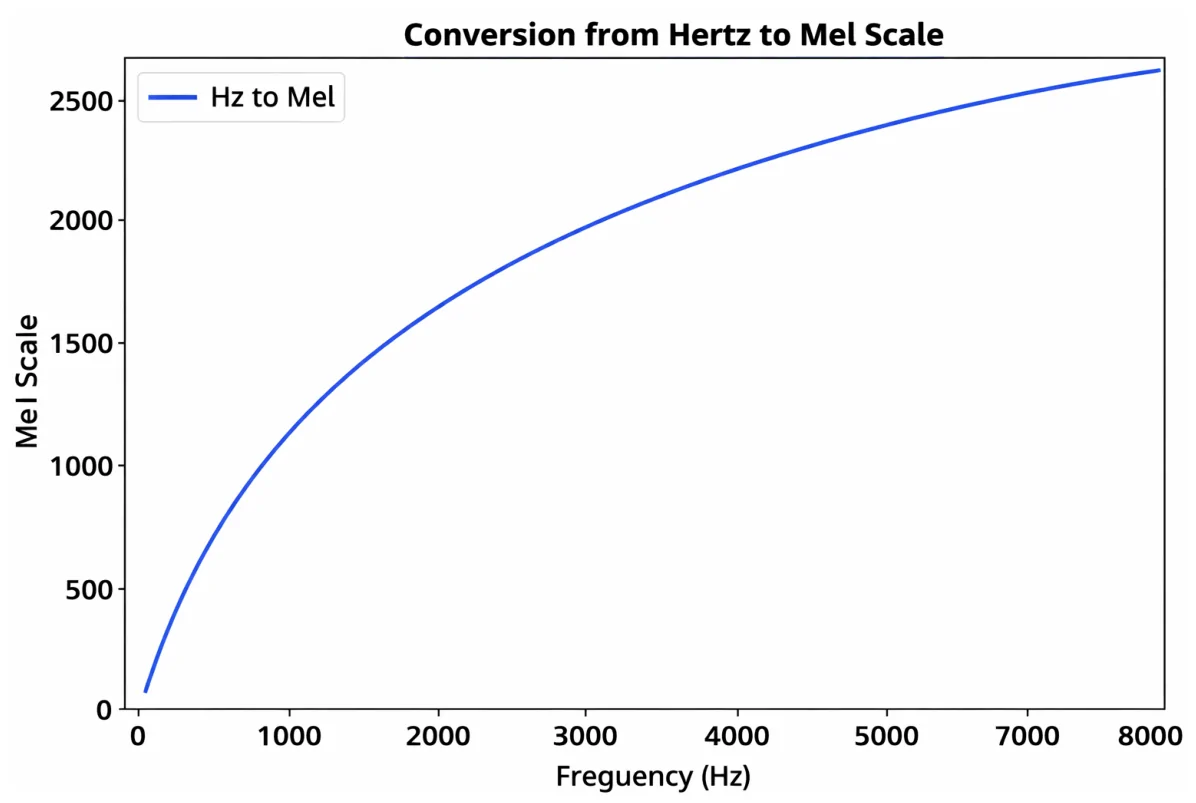
Illustration of the nonlinear Hertz-to-Mel frequency mapping.

Figure 6 illustrates this perceptual frequency warping, where lower-frequency components are expanded and higher-frequency components are compressed. A Mel spectrogram extends this concept by representing the time–frequency energy distribution of a signal on the Mel scale. Compared with conventional linear-frequency spectrograms, Mel spectrograms provide enhanced resolution in perceptually relevant frequency bands and serves as the basis for extracting compact cepstral representations such as MFCCs [33]. In the context of neural signal analysis, MFCCs provide a compact cepstral representation of the spectral envelope while preserving discriminative temporal variations across successive analysis windows. Recent studies have demonstrated the effectiveness of MFCC-based cepstral representations for biomedical signal analysis. Wei Pei et al. [62] showed that Mel-frequency cepstral coefficients extracted from EEG and EMG signals, when structured as two-dimensional representations and processed using a CNN–LSTM architecture, significantly improved sleep stage neural decoding performance. Extending MFCC-based analysis to cardiac signals, Toulni et al. [63] reported that cepstral descriptors derived from wavelet-decomposed ECG signals enabled accurate neural decoding using support vector machines. Furthermore, the comprehensive review by Abdul and Al-Talabani [33] highlighted the broad applicability of cepstral descriptors across diverse biomedical modalities, emphasizing their robustness and discriminative capability for physiological signal neural decoding and neural event detection. These studies indicate that MFCC-based representations are not limited to speech signals and can be effectively transferred to neural signal domains.

**Figure 7:**
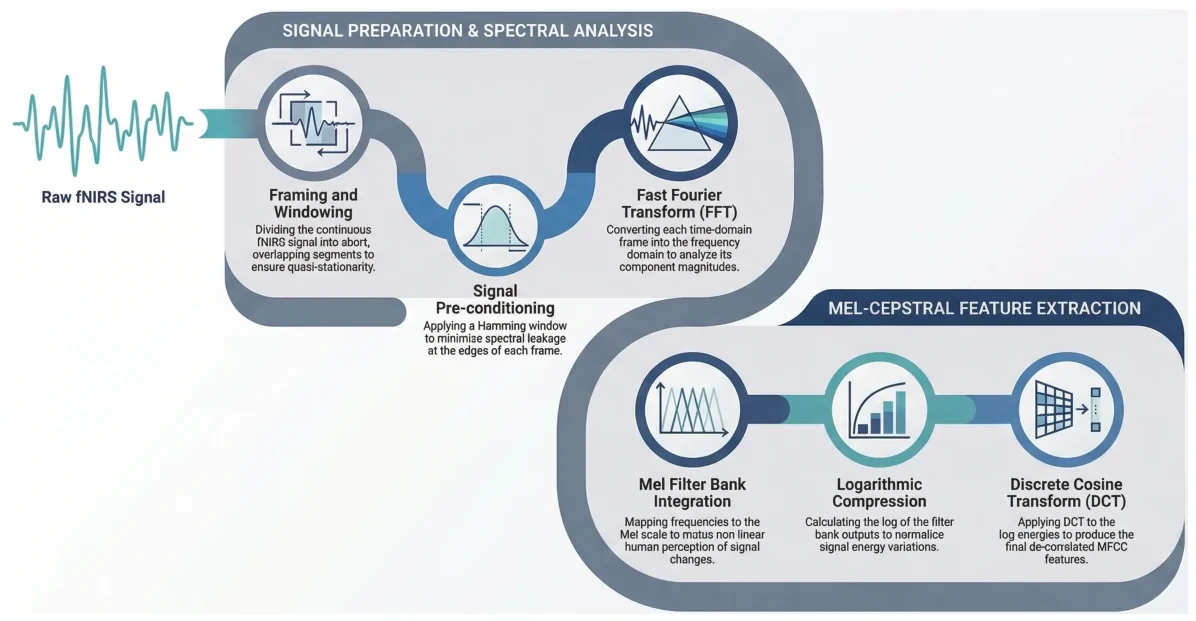
MFCC extraction pipeline.

Motivated by these findings, the present study extends the application of MFCCs to functional near-infrared spectroscopy (fNIRS) signals. The adapted pipeline for the extraction of MFCCs is shown in Figure 7. Unlike speech signals, fNIRS hemodynamic signals are characterized by low-frequency spectral components; therefore, the conventional MFCC procedure was adapted to the frequency characteristics of the fNIRS signal. The extraction process consists of framing and windowing the preprocessed HbO signal, frequency-domain transformation, Mel filter-bank analysis, logarithmic compression, and discrete cosine transformation (DCT), resulting in a compact cepstral representation of the hemodynamic signal.

Because the 10 s task execution window (125 samples) is shorter than the nominal 128-sample frame length, framing and MFCC extraction were applied continuously to the preprocessed session-level HbO signal prior to trial segmentation. Let x[n] denote the continuous discrete-time fNIRS signal for a given session, sampled at $f_{s}=12.5$ Hz. A frame length of $N_{w}=128$ samples was used, corresponding to a frame duration of

(7) $$T_{w}=\frac{N_{w}}{f_{s}}=\frac{128}{12.5}=10.24\;s.$$

The *m*-th frame was obtained by shifting the signal by the hop size $N_{h}$ and applying a Hamming window:

(8) $$x_{m}\left[n\right]=x\left[n+mN_{h}\right]\;w\left[n\right],\;0\leq n<N_{w},$$

where $x_{m}\left[n\right]$ denotes the *m*-th windowed frame, $N_{w}$ is the frame length in samples, $N_{h}$ is the hop size, and w[n] is the analysis window. A 50% overlap was used between consecutive frames, such that

(9) $$N_{h}=\frac{N_{w}}{2}=64\;\mathrm{samples},$$

corresponding to a hop duration of

(10) $$T_{h}=\frac{N_{h}}{f_{s}}=\frac{64}{12.5}=5.12\;s.$$

Thus, each frame spans 10.24 s and consecutive frames are separated by 5.12 s. A Hamming window was employed to reduce spectral leakage and is defined as

(11) $$w\left[n\right]=0.54-0.46\cos\left(\frac{2\pi n}{N_{w}-1}\right).$$

Zero padding was applied where necessary to ensure a uniform number of samples for each frame.

After framing and windowing, the frequency-domain representation of each frame was obtained using the discrete Fourier transform (DFT), implemented using the fast Fourier transform (FFT). For the *m*-th frame, the transform is expressed as

(12) $$X_{m}\left[k\right]=\sum_{n=0}^{N_{w}-1}x_{m}\left[n\right]e^{-j2\pi kn/N_{\mathrm{FFT}}},$$

where $X_{m}\left[k\right]$ is the complex spectrum of frame *m*, *k* is the frequency-bin index, and $N_{\mathrm{FFT}}$ is the number of DFT points. To ensure sufficient frequency resolution within the narrow 0.01–0.3 Hz hemodynamic passband, the 128-sample frames were zero-padded to $N_{\mathrm{FFT}}=1024$ points prior to the DFT. At a sampling frequency of 12.5 Hz, this provides a frequency resolution of Δf=12.5/1024≈0.012 Hz per bin, yielding an adequate number of discrete frequency bins to support the subsequent 26-filter Mel bank.

The corresponding power spectrum was calculated as

(13) $$P_{m}\left[k\right]=\frac{1}{N_{\mathrm{FFT}}}{\left|X_{m}\left[k\right]\right|}^{2}.$$

**Figure 8:**
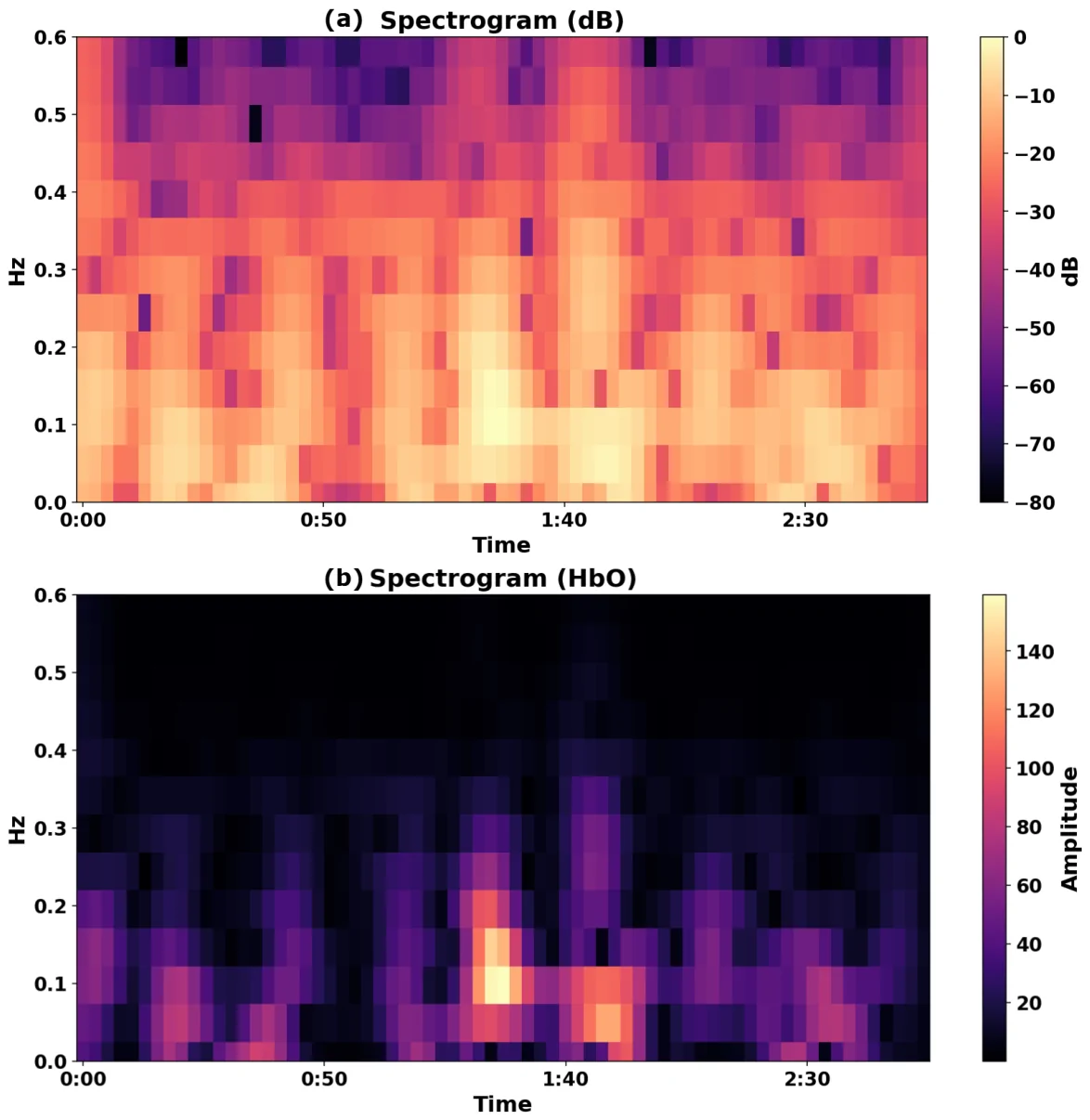
Time–frequency representations of the fNIRS signal. (**a**) Vanilla spectrogram, showing the spectral energy distribution across frequency and time. Although stronger energy is observed at lower frequencies, the frequency range used for subsequent spectral feature analysis was restricted to 0.01–0.3 Hz. (**b**) Corresponding HbO spectrogram illustrating the temporal variation of HbO spectral amplitude within the displayed frequency range.

The resulting power spectrum describes the distribution of signal energy across frequency for each individual frame.The power spectra obtained from the individual frames were combined to form a time-frequency representation of the fNIRS signal, referred to as the vanilla spectrogram. The vanilla spectrogram represents the temporal evolution of spectral power across the frequency range and provides a visual representation of changes in spectral energy between task and resting conditions as illustrated in Figure 8a.

The power spectrum was subsequently mapped to the Mel-frequency scale using a bank of triangular filters. The Hertz-to-Mel transformation is defined as

(14) $$\mathrm{mel}\left(f\right)=1127\ln\left(1+\frac{f}{700}\right),$$

where *f* denotes frequency in Hertz.

For the present fNIRS application, the filter bank consisted of J=26 triangular filters spanning the frequency range 0.01–0.3 Hz.

The response of the *j*-th Mel filter is defined as

(15) $$H_{j}\left(f\right)=\left\{\begin{array}{cc} 0, & f<f_{j-1},\\ \frac{f-f_{j-1}}{f_{j}-f_{j-1}}, & f_{j-1}\leq f\leq f_{j},\\ \frac{f_{j+1}-f}{f_{j+1}-f_{j}}, & f_{j}\leq f\leq f_{j+1},\\ 0, & f>f_{j+1}, \end{array}\right.$$

where j=1,2,…,J denotes the Mel filter index, while $f_{j-1}$, $f_{j}$, and $f_{j+1}$ represent the lower, center, and upper frequencies of the *j*-th triangular filter, respectively. The energy of the *j*-th Mel filter for frame *m* was then obtained by weighting the power spectrum with the corresponding filter:

(16) $$S_{m}\left[j\right]=\sum_{k}P_{m}\left[k\right]H_{j}\left[k\right],\;j=1,2,\ldots ,J,$$

where $P_{m}\left[k\right]$ is the power spectrum of frame *m* and $H_{j}\left[k\right]$ is the frequency-domain response of the *j*-th Mel filter. Because fNIRS signals occupy a very low-frequency range compared with speech signals, the Hertz-to-Mel transformation is approximately linear within the frequency range considered in this study. Consequently, the resulting Mel filters are close to uniformly spaced in Hertz over the 0.01–0.3 Hz range. Therefore, the present study does not assume that the nonlinear Mel-frequency warping itself provides a specific physiological advantage for fNIRS signals. Instead, the filter-bank stage is used primarily to aggregate neighboring spectral components into a compact set of frequency-band energies. The subsequent logarithmic compression and discrete cosine transformation (DCT) further compress and decorrelate these filter-bank energies, yielding a low-dimensional representation of the spectral envelope. Thus, in the present application, the term MFCC refers to the complete filter-bank, logarithmic-compression, and DCT-based cepstral representation rather than to a claim that Mel-frequency spacing is intrinsically superior to linear frequency spacing for fNIRS.

**Figure 9:**
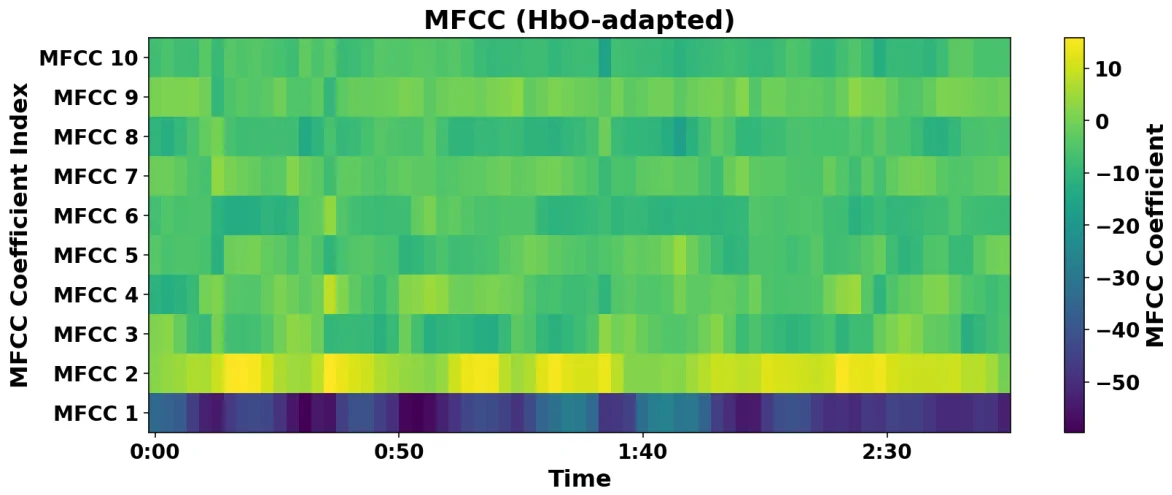
MFCCs of one trial from Subject 1.

The Mel filter-bank energies obtained across successive frames form the Mel spectrogram, which provides a time-frequency representation after mapping the spectral energy to the Mel scale. Figure 8b illustrates the resulting Mel spectrogram for the rest and task conditions of Subject 1. The Mel filter-bank energies were subsequently transformed using logarithmic compression:

(17) $$L_{m}\left[j\right]=\log\left(S_{m}\left[j\right]+\epsilon \right),$$

where $L_{m}\left[j\right]$ represents the log Mel energy for filter *j* in frame *m*, and ϵ is a small positive constant introduced to prevent numerical instability when $S_{m}\left[j\right]$ approaches zero. The logarithmic transformation compresses the dynamic range of the filter-bank energies and provides a more compact representation of the spectral-energy distribution. Finally, the DCT was applied to the log Mel filter-bank energies to decorrelate the spectral representation and obtain the MFCC coefficients. The *c*-th MFCC coefficient for frame *m* is defined as

(18) $${\mathrm{MFCC}}_{m}\left[c\right]=\sum_{j=1}^{J}L_{m}\left[j\right]\cos\left[\frac{\pi c}{J}\left(j-\frac{1}{2}\right)\right],\;c=0,1,\ldots ,C-1,$$

where *c* denotes the MFCC coefficient index, J=26 is the number of Mel filters, and *C* is the number of retained MFCC coefficients. In the present study, the first 10 MFCC coefficients were retained, resulting in a C=10-dimensional cepstral vector per frame. Higher-order coefficients primarily represent rapid variations in the spectral envelope and are more susceptible to noise in band-limited hemodynamic signals. The first 10 coefficients were therefore retained as a compact representation of the dominant spectral characteristics. This differs from the earlier study [34], in which 13 coefficients were retained. The reduction to 10 coefficients in the present study was based on the observation that coefficients beyond the tenth did not provide measurable improvement on the training folds while increasing the dimensionality of the fused feature representation. Repeating the above procedure for all *M* frames produces the MFCC feature matrix

(19) $$M\in R^{C\times M}$$

where C=10 denotes the number of retained MFCC coefficients and *M* denotes the total number of frames. Each column corresponds to one temporal frame, while each row corresponds to one MFCC coefficient. Following continuous feature extraction across the session, trial segmentation was performed. For each trial, only MFCC frames whose temporal centers fell within the 10 s task execution window were retained and averaged to obtain a single 10-dimensional cepstral feature vector for each channel/trial. No additional temporal margin was applied. Thus, frames whose temporal centers fell outside the task execution window, including those centered in the inter-trial rest period, were excluded from the trial-level MFCC representation. This zero-margin selection was used to prevent the inclusion of rest-period frames and thereby minimize potential label contamination between adjacent trial conditions. These resulting MFCC descriptors constitute the cepstral branch of the proposed framework and are shown in Figure 9.

In the context of fNIRS, task-related hemodynamic responses exhibit characteristic low-frequency spectral patterns associated with neural activation. By representing these patterns through MFCC-based cepstral descriptors, complementary spectral information is captured that is not explicitly encoded by temporal hemodynamic descriptors alone. Since the temporal and cepstral representations characterize different but complementary properties of the same hemodynamic response, their adaptive integration provides a more comprehensive feature representation for neural decoding. Accordingly, the extracted MFCC coefficients are combined with the temporal hemodynamic descriptors through the proposed Adaptive Multi-Domain Feature Fusion (AMFF) framework, which is described in the following subsection.

### 2.6. Adaptive Multi-Domain Feature Fusion (AMFF)

Temporal hemodynamic descriptors and MFCC-based cepstral descriptors characterize complementary properties of fNIRS signals. Temporal descriptors summarize the statistical behavior of the hemodynamic response in the time domain, whereas MFCC-based cepstral descriptors provide a compact representation of task-related spectral patterns. Conventional feature fusion strategies typically concatenate these heterogeneous feature sets into a single feature vector before classification. Although computationally simple, direct concatenation implicitly assumes that both feature domains contribute equally to neural decoding, despite their discriminative importance varying across cognitive tasks, subjects, and recording conditions.

**Figure 10:**
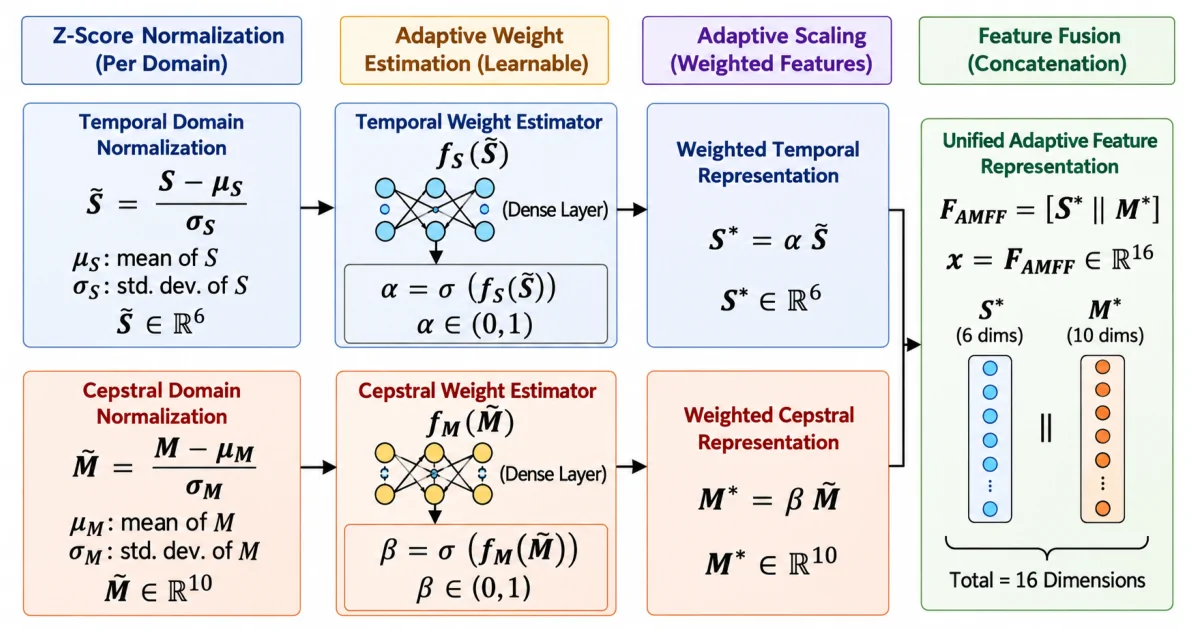
Adaptive multi-domain feature fusion (AMFF).

To overcome this limitation, the proposed Adaptive Multi-Domain Feature Fusion (AMFF) framework introduces a lightweight trainable weighting mechanism that adaptively learns the relative importance of the temporal and cepstral feature domains during network training (Figure 10). Instead of assigning equal importance to temporal and cepstral information, AMFF allows the neural network to emphasize the more informative domain while suppressing less discriminative information. Consequently, the fused representation becomes more robust and better suited for four-class neural decoding. Let

(20) $$S={\left[s_{1},s_{2},\ldots ,s_{6}\right]}^{T}$$

represent the temporal hemodynamic descriptor vector, where $s_{1},\cdots ,s_{6}$ correspond to the six statistical descriptors extracted from the fNIRS signal. Similarly,

(21) $$M={\left[m_{1},m_{2},\cdots ,m_{10}\right]}^{T}$$

denotes the spectral descriptor vector consisting of the ten extracted MFCC coefficients. Since the statistical descriptors and MFCC coefficients have different numerical ranges and statistical distributions, each feature domain is independently normalized using z-score normalization before adaptive weighting. This prevents features with larger numerical magnitudes from dominating the optimization process and ensures comparable feature scales during learning. The normalized feature vectors are computed as

(22) $$\tilde{S}=\frac{S-\mu_{S}}{\sigma_{S}},$$

and

(23) $$\tilde{M}=\frac{M-\mu_{M}}{\sigma_{M}},$$

where $\mu_{S}$ and $\sigma_{S}$ denote the mean and standard deviation of the temporal feature domain, while $\mu_{M}$ and $\sigma_{M}$ represent the corresponding statistics of the spectral feature domain. To avoid information leakage across the leave-one-subject-out folds, these normalization statistics were estimated exclusively from the 28 training subjects of each fold and then applied unchanged to the held-out subject. After normalization, AMFF estimates the relative importance of each feature domain through two independent adaptive weighting functions. These weights are generated by lightweight trainable dense layers followed by sigmoid activation functions, allowing the weighting coefficients to be optimized jointly with the classifier during network training. The adaptive weights are expressed as

(24) $$\alpha =\sigma \;\left(f_{S}\left(\tilde{S}\right)\right),$$

(25) $$\beta =\sigma \;\left(f_{M}\left(\tilde{M}\right)\right),$$

where $f_{S}\left(\;\cdot \;\right)\;:R^{6}\;\to \;R$ and $f_{M}\left(\;\cdot \;\right)\;:R^{10}\;\to \;R$ denote trainable fully connected layers with scalar output and σ(·) represents the sigmoid activation function. Consequently, both adaptive weights are scalars constrained to the interval [0,1], enabling the network to regulate the contribution of each feature domain according to its discriminative relevance. It is important to note that α and β are functions of the input feature vectors themselves and are therefore evaluated per sample rather than being learned as fixed constants. This distinction matters: a pair of fixed scalar gains applied to two concatenated blocks could be absorbed into the first weight matrix of the subsequent classifier and would add no representational capacity, whereas input-dependent gating allows the relative emphasis placed on the two domains to vary with the observation being decoded. In this respect AMFF belongs to the family of gated, or squeeze-and-excitation style, fusion mechanisms, and is distinguished from them by its lightweight two-branch form and by operating on handcrafted rather than learned representations. The normalized feature vectors are then scaled using their corresponding adaptive weights,

(26) $$S^{*}=\alpha \tilde{S},$$

(27) $$M^{*}=\beta \tilde{M}.$$

This adaptive scaling allows the network to strengthen informative feature domains while reducing the influence of less discriminative representations. Unlike conventional concatenation, where all features contribute equally, the proposed weighting mechanism enables dynamic feature balancing during optimization. Finally, the weighted temporal and spectral feature representations are concatenated to generate the unified adaptive feature representation,

(28) $$F_{\mathrm{AMFF}}=\left[S^{*}\;\left|\right|\;M^{*}\right].$$

The resulting feature vector is subsequently provided as the input to the multilayer perceptron (MLP) neural decoder. Because the adaptive weighting parameters are learned simultaneously with the MLP classifier through backpropagation, the proposed AMFF framework performs end-to-end optimization without introducing significant computational overhead. This enables the network to automatically determine the optimal balance between temporal hemodynamic descriptors and spectral MFCC descriptors, thereby improving the discriminative capability of the fused representation for multi-class fNIRS neural decoding.

### 2.7. Neural Decoding

The unified adaptive feature representation generated by the proposed Adaptive Multi-Domain Feature Fusion (AMFF) framework is classified using a multilayer perceptron (MLP) neural decoder. MLPs are widely employed in fNIRS-based brain–computer interface (BCI) systems because of their ability to model nonlinear relationships between handcrafted feature representations and cognitive states [16,54].

In this study, a four-layer multilayer perceptron (MLP) architecture was employed, comprising an input layer, two hidden layers with 25 neurons each, and an output layer. The relatively compact architecture was motivated by the low-dimensional handcrafted feature representation, which comprised six temporal descriptors and ten MFCC coefficients. A larger network was not considered necessary for modeling the resulting 16-dimensional representation and could introduce additional parameters without a corresponding increase in the available subject-level training information. The hidden-layer depth and width were determined through an inner validation procedure performed independently within each leave-one-subject-out (LOSO) fold, using only the training subjects of that fold. Candidate MLP architectures were evaluated on an inner validation subset, while the held-out subject was completely excluded from architecture selection. The inner hyperparameter search was therefore repeated independently for each LOSO fold. The same two-hidden-layer architecture with 25 neurons per layer was selected in all folds and was subsequently fixed before evaluation on the corresponding held-out subject.

The weighted temporal hemodynamic and MFCC-based cepstral descriptors generated by the AMFF framework are concatenated to form the unified adaptive feature representation, which serves as the input to the MLP neural decoder.

(29) $$x=F_{\mathrm{AMFF}}=\left[S^{*}\;\left|\right|\;M^{*}\right]\in R^{16},$$

where x denotes the unified adaptive feature representation generated by the proposed AMFF framework. One such 16-dimensional vector constitutes a single classification sample, obtained from each trial by extracting features from the preprocessed HbO signal across the 10 s task execution window and averaging the feature vectors across the 28 measurement channels. This channel-level aggregation yields one 16-dimensional trial representation and therefore 120 trial samples per participant (30 samples per class across the four task conditions).

The 28 measurement channels were aggregated at the feature level by averaging the extracted feature vectors across channels for each trial. This strategy was adopted to obtain a compact trial-level representation while preserving the overall hemodynamic response captured across the measurement montage. Specifically, averaging reduces channel-specific variability and prevents the feature dimensionality from increasing from 16 to 28×16=448 dimensions per trial, which would substantially increase the number of trainable parameters relative to the limited number of independent subjects available for subject-independent evaluation. This design is also consistent with the primary objective of the present study, which is to investigate the complementary contribution of temporal and cepstral feature domains rather than to model channel-specific spatial patterns. Importantly, channel averaging does not mix trials or participants; aggregation is performed only among the 28 channels belonging to the same trial after feature extraction. A separate channel-wise feature representation was not evaluated in the present study. Therefore, the present results should be interpreted as reflecting multi-channel aggregated hemodynamic information rather than channel-specific spatial decoding.

Samples were assigned to folds strictly by subject, and no two samples originating from the same trial were split across training and validation, so that neither subject identity nor within-trial temporal correlation could leak between the two. The transformation at the first hidden layer is given by,

(30) $$h_{1}=\tanh\left(W_{1}x+b_{1}\right),$$

where $W_{1}\in R^{25\times 16}$ and $b_{1}\in R^{25}$ denote the weight matrix and bias vector, respectively. The hyperbolic tangent activation function introduces nonlinearity, enabling the network to model complex relationships between the fused temporal and spectral feature representations.The second hidden layer output is computed as

(31) $$h_{2}=\tanh\left(W_{2}h_{1}+b_{2}\right),$$

where $W_{2}\in R^{25\times 25}$ and $b_{2}\in R^{25}$. The output layer produces the class scores according to

(32) $$z=W_{3}h_{2}+b_{3},$$

where $W_{3}\in R^{C\times 25}$ and $b_{3}\in R^{C}$, with *C* denoting the number of target classes. For multi-class neural decoding, the predicted class probabilities are obtained using the softmax function. The Softmax activation converts the output logits into a normalized probability distribution across the four cognitive states, and the class with the highest probability is selected as the predicted label.

(33) $${\hat{y}}_{c}=\frac{e^{z_{c}}}{\sum_{j=1}^{C}e^{z_{j}}},\;c=1,2,\ldots ,C.$$

The network parameters $\left\{W_{l},b_{l}\right\}$ were optimized by minimizing the categorical cross-entropy loss function

(34) $$L=-\sum_{c=1}^{C}y_{c}\log\left({\hat{y}}_{c}\right),$$

where $y_{c}$ represents the ground-truth class label. Minimizing the categorical cross-entropy loss encourages the predicted probability distribution to match the ground-truth class labels, thereby improving the discriminative capability of the neural decoder. Training was performed end-to-end using the limited-memory Broyden–Fletcher–Goldfarb–Shanno (L-BFGS) optimization algorithm. The L-BFGS step-size was set to 1.0 and the maximum number of iterations per optimization step was 25. The optimizer used the default PyTorch convergence tolerances and history size. L-BFGS was selected because the proposed neural decoder is intentionally lightweight and operates on a low-dimensional handcrafted feature representation. Specifically, the MLP consists of 16 input features, two hidden layers with 25 neurons each, and a four-class output layer, while the AMFF weighting components add only a small number of additional trainable parameters. This relatively small optimization problem makes a quasi-Newton method such as L-BFGS suitable for deterministic full-batch optimization, where curvature information can be exploited to accelerate convergence. In contrast, stochastic first-order optimizers such as Adam and SGD are more commonly employed for large-scale neural networks and mini-batch training. During optimization, the parameters of both the proposed AMFF framework and the MLP classifier were updated simultaneously through backpropagation, allowing the adaptive weighting mechanism to learn the relative contribution of the temporal and cepstral hemodynamic descriptors [64,65].

The framework was implemented in PyTorch 2.2.0, and the architectural choices (two hidden layers of 25 units, ten retained MFCCs, twenty-six Mel filters) were fixed by means of an inner validation split constructed from the training subjects of each fold only, so that no held-out subject influenced model selection.

## 3. Results

This section evaluates the proposed multi-domain fNIRS-BCI framework against its temporal-only and cepstral-only counterparts. We proceed from evidence to inference: subject-wise results establish the core performance parameters trend (Section 3.3); group-level and distributional views confirm this trend is consistent rather than driven by outliers (Section 3.3 continued); statistical tests then establish whether the observed differences are significant (Section 3.4 and Section 3.5); MFCC parameter selection analysis (Section 3.6); analysis of weight alpha and beta in AMFF (Section 3.7) and a final ablation isolates which individual temporal descriptors drive the fusion benefit (Section 3.8).

### 3.1. Evaluation Setup

To evaluate the neural decoding performance of the proposed fNIRS-BCI framework, a subject-independent leave-one-subject-out (LOSO) cross-validation strategy was employed. The complete dataset comprised 29 subjects, and the subject, rather than individual trials or samples, was used as the unit of partitioning. In each of the 29 LOSO iterations, data from 28 subjects were used exclusively for model training, while all data from the remaining subject were held out for validation. The procedure was repeated until every subject had served exactly once as the held-out validation subject. Thus, no samples, trials, or feature vectors from the validation subject were included in the corresponding training set, thereby preventing subject-specific information from being shared between training and validation and providing an assessment of the model’s ability to generalize across previously unseen individuals. This subject-independent evaluation is particularly important for fNIRS-based BCIs because substantial inter-subject variability can affect the characteristics of hemodynamic responses and consequently influence neural decoding performance.

All experiments were conducted on a workstation running Ubuntu 24.04 LTS, equipped with an Intel Core i9-14900K CPU and 128 GB RAM. Because the proposed network is small and is trained with a quasi-Newton solver, training and inference were executed on the CPU and no GPU acceleration was required. Model performance was assessed using standard evaluation metrics, including accuracy, precision, recall (sensitivity), and F1-score. With four balanced classes, the chance level for accuracy is 25%.

### 3.2. Evaluation Metrics

Evaluation metrics play a critical role in assessing the effectiveness of machine learning models, particularly in multi-class neural decoding tasks. The confusion matrix categorizes predictions into true positives (TP), false positives (FP), true negatives (TN), and false negatives (FN). Based on these quantities, commonly used performance metrics were computed as follows:

(35) $$Accuracy=\frac{TP+TN}{TP+TN+FP+FN}$$

(36) $$Precision\;\left(PPV\right)=\frac{TP}{TP+FP}$$

(37) $$Recall\;\left(TPR\right)=\frac{TP}{TP+FN}$$

(38) $$F1\text{-}score=2\;\cdot \;\frac{Precision\;\cdot \;Recall}{Precision+Recall}$$

For the four-class neural decoding task (LHMI, RHMI, MA, Rest), each metric was computed per class using a one-vs-rest scheme and macro-averaged across classes.

### 3.3. Subject-Wise Neural Decoding Performance

Having established the evaluation protocol, we first evaluate the extent to which combining temporal and cepstral information improves neural decoding compared with either domain alone. In addition, to distinguish the benefit of multi-domain feature integration from the specific contribution of adaptive fusion, a simple concatenation baseline is included, in which temporal and cepstral descriptors are directly concatenated before classification without adaptive weighting. This allows the proposed Adaptive Multi-domain Feature Fusion (AMFF) to be evaluated against both single-domain representations and a conventional non-adaptive fusion strategy. Table 2 presents accuracy, precision, recall, and F1-score for all four feature representations across all 29 participants.

The table confirms a consistent improvement from single-domain to multi-domain representations across the 29 participants. Mean accuracy increases from 90.7% for temporal features to 95.0% for cepstral features, 95.2% for simple concatenation, and 95.6% for the proposed AMFF representation. Thus, directly concatenating temporal and cepstral descriptors already provides a small improvement over the cepstral representation alone, while AMFF provides a further gain of 0.4 percentage points over simple concatenation. Importantly, the comparison between the two multi-domain representations isolates the contribution of the adaptive fusion mechanism because both use the same temporal and cepstral feature domains and the same MLP classifier, differing primarily in how the two feature domains are integrated.

The benefit of AMFF over simple concatenation is consistent across all four evaluation metrics. AMFF improves accuracy from 95.2% to 95.6%, precision from 94.2% to 94.6%, recall from 95.4% to 95.9%, and F1-score from 94.9% to 95.3%. The corresponding mean improvements are 0.38, 0.41, 0.50, and 0.43 percentage points, respectively. Notably, the largest additional improvement occurs in recall, suggesting that adaptive fusion provides a greater benefit in preserving discriminative information relevant to correctly identifying the target neural states than direct concatenation. In contrast, the relatively small difference between concatenation and AMFF indicates that most of the substantial improvement over the temporal baseline originates from incorporating complementary cepstral information, while AMFF provides an additional, consistent refinement of this combined representation.

At the individual-subject level, AMFF achieves higher accuracy than simple concatenation for all 29 participants, with the improvement ranging from 0.2 to 0.7 percentage points. The same consistent pattern is observed for precision, recall, and F1-score, for which AMFF also exceeds simple concatenation for all 29 subjects. Therefore, the improvement associated with AMFF is not attributable to a small number of high-performing participants; rather, the adaptive fusion strategy provides a small but systematic benefit over direct feature concatenation across the subject population.

Beyond accuracy, Table 2 also reveals a precision-recall asymmetry across feature representations. Temporal descriptors yield substantially higher precision than recall on average (93.1% vs. 89.2%), indicating a tendency toward missed detections with temporal features alone. In contrast, cepstral, concatenated multi-domain, and AMFF representations all shift toward higher recall than precision. For the cepstral representation, recall exceeds precision by 1.9 percentage points, while the corresponding differences are 1.2 and 1.3 percentage points for concatenation and AMFF, respectively. Thus, incorporating cepstral information changes the balance between precision and recall, while adaptive fusion further improves both measures relative to direct concatenation, with the largest additional gain observed in recall.

**Figure 11:**
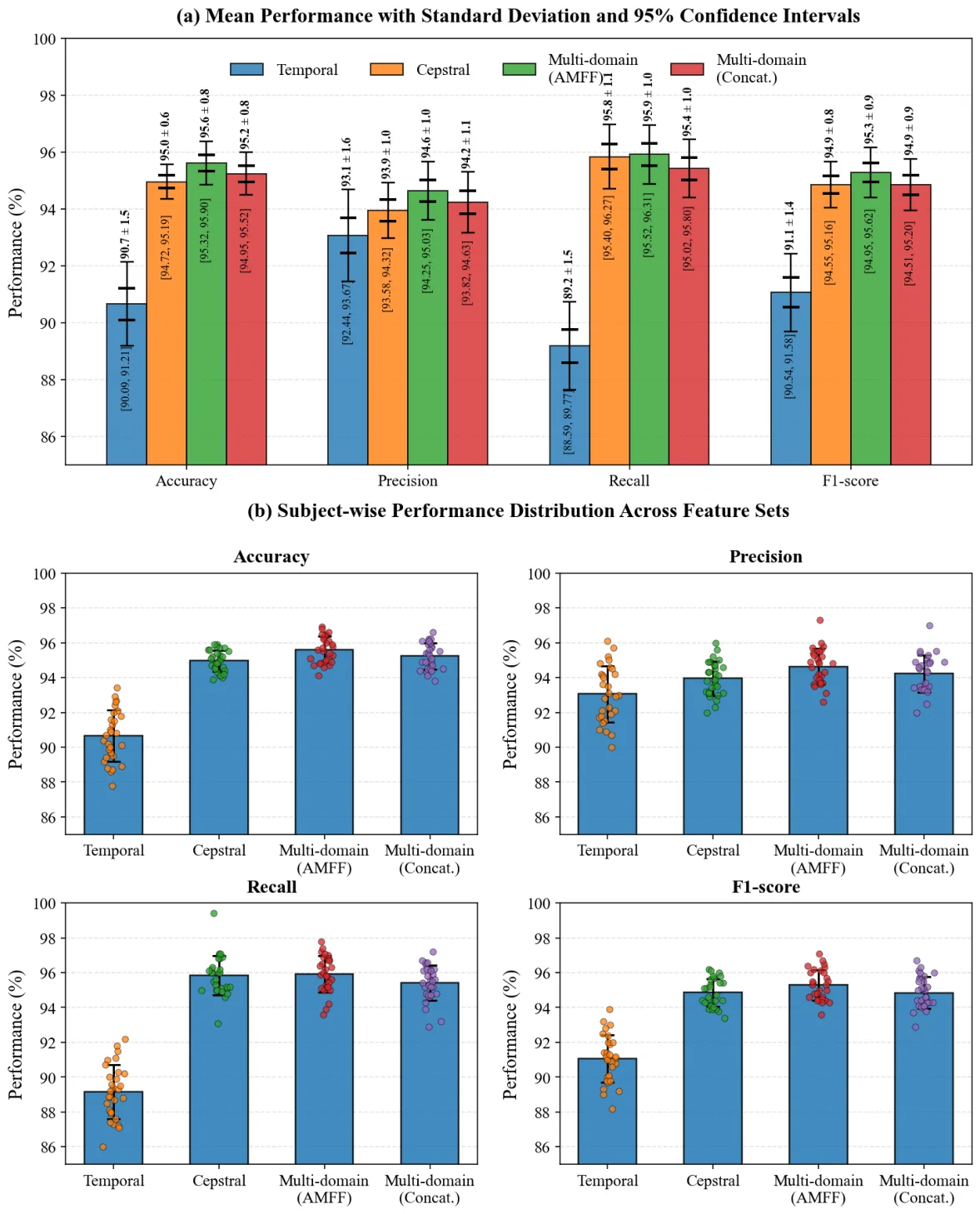
Average classification performance (mean ± standard deviation) and subject-wise distribution across the four feature representations for 29 subjects. (**a**) Mean performance for accuracy, precision, recall, and F1-score using temporal, cepstral, multi-domain concatenation, and multi-domain AMFF representations; (**b**) subject-wise distribution of accuracy, precision, recall, and F1-score, where bars represent the mean performance and individual markers indicate the results obtained for each subject.

This subject-level pattern is summarized at the group level in Figure 11a, which aggregates the same 29-subject data reported in Table 2. The mean values reproduce the accuracy hierarchy observed per subject and show a clear progression from temporal to cepstral and multi-domain representations, while also revealing the incremental contribution of adaptive fusion. Mean accuracy increases from 90.7% for temporal features to 95.0% for cepstral features, 95.2% for direct concatenation, and 95.6% for AMFF. The relatively small difference between the concatenation and AMFF representations is nevertheless consistent across all four metrics, with AMFF providing additional gains in accuracy, precision, recall, and F1-score. The accompanying standard deviations, reported throughout as the population standard deviation across the 29 subjects, quantify the variability across subjects and reflect the variability reduction already visible in the table’s spread. Standard deviation drops sharply between temporal and cepstral features (e.g., accuracy SD: 1.45→0.60), reflecting a substantial reduction in inter-subject variability once spectral information is introduced. This reduced variability is largely maintained, rather than further reduced, by the multi-domain representations: the concatenation representation exhibits an accuracy SD of 0.74, while AMFF yields an SD of 0.75, and SD values for precision and F1-score remain close to those of the cepstral features. Recall is the only metric for which variability continues to decrease under multi-domain fusion, decreasing from 1.12 for cepstral features to 1.01 for concatenation and 1.02 for AMFF. Together with Table 2, these results indicate that the largest gain in cross-subject consistency comes from incorporating spectral descriptors, with the multi-domain representations preserving this consistency while additionally improving mean performance. The relatively small difference between AMFF and direct concatenation therefore represents an additional improvement in mean performance rather than a further systematic reduction in inter-subject variability.

The mean-and-standard-deviation summary in Figure 11a provides an overall view of performance variability, but it does not reveal the underlying distribution of individual subject results. Figure 11b addresses this by plotting every subject’s value alongside the group mean for all four performance metrics. Consistent with the narrower ranges already observed in Table 2, the individual data points for the cepstral and multi-domain feature representations form visibly tighter clusters around their respective means across all four panels, with no evidence of a bimodal distribution or isolated outlier subjects disproportionately influencing the mean. These findings further support the earlier observation that the improvement in classification performance is broadly shared across participants rather than being driven by a small subset of high-performing individuals. In conjunction with the consistent subject-wise improvement of AMFF over direct concatenation, the distribution analysis indicates that the additional benefit of adaptive fusion is not attributable to a small number of individual subjects, but is observed across the participant population.

**Figure 12:**
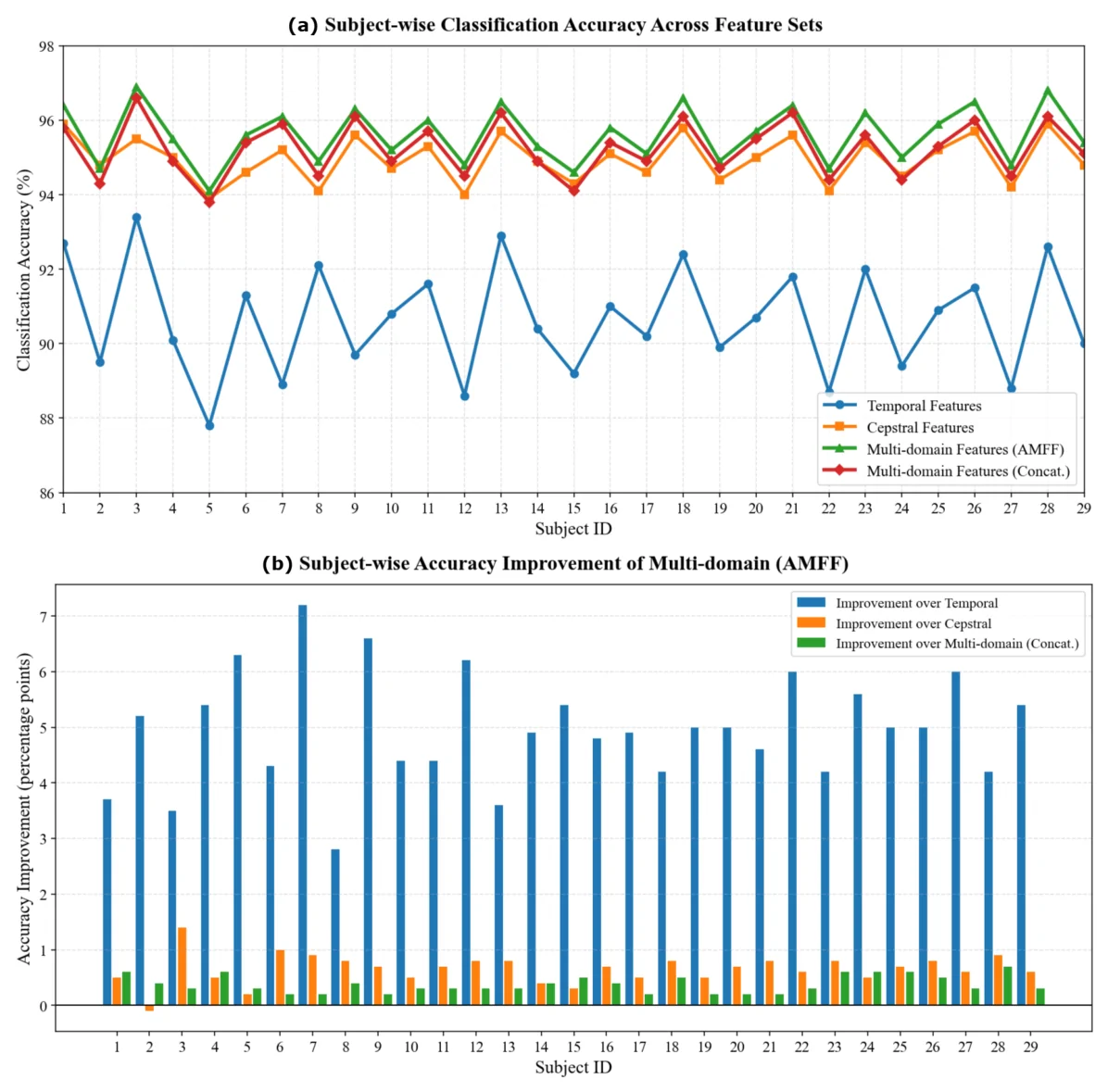
(**a**) Subject-wise classification accuracy obtained using temporal, cepstral, multi-domain concatenation, and multi-domain AMFF feature representations; (**b**) subject-wise accuracy improvement obtained from multi-domain representations relative to the corresponding single-domain baselines, including the additional improvement of AMFF over simple concatenation.

To examine this consistency at the level of individual participants directly, Figure 12a plots per-subject accuracy for the four feature representations. Temporal features remain the lowest-performing representation for every subject, demonstrating the consistent benefit of incorporating cepstral information. Direct concatenation further improves upon the cepstral representation for most participants, although the difference is small and not uniform across all subjects. In contrast, AMFF achieves higher accuracy than simple concatenation for all 29 participants, with an improvement ranging from 0.2 to 0.7 percentage points. This consistent subject-level advantage provides direct evidence that the performance gain associated with AMFF is not solely due to combining temporal and cepstral features, but also reflects the benefit of adaptively integrating the two feature domains. This near-perfect rank consistency reinforces that the hierarchy established in Table 2 is not an artifact of averaging, and it is precisely what the concordance coefficient reported in Section 3.4 quantifies.

Having established that the ranking of feature representations is consistent across subjects, Figure 12b further quantifies the magnitude of the performance improvement obtained from the multi-domain representations and examines whether these gains vary according to a subject’s baseline performance with the single-domain feature representations. Relative to temporal descriptors, the AMFF representation improves accuracy by an average of 5.0 percentage points, with individual gains ranging from 2.8 percentage points (Subject 8) to 7.2 percentage points (Subject 7). Direct concatenation produces a comparable overall improvement over temporal features, reflecting the substantial contribution of the complementary cepstral information. In contrast, the improvement of AMFF over cepstral descriptors is substantially smaller, averaging approximately 0.7 percentage points (range: −0.1 to +1.4 percentage points), consistent with the earlier observation that cepstral features already capture most of the discriminative information. Importantly, AMFF provides a further mean improvement of 0.38 percentage points over direct concatenation, with positive gains of 0.2–0.7 percentage points across all 29 subjects. The same pattern is observed across the other performance measures: compared with direct concatenation, AMFF provides mean improvements of 0.41 percentage points in precision, 0.50 percentage points in recall, and 0.43 percentage points in F1-score. The larger improvement in recall indicates that the adaptive fusion mechanism is particularly beneficial for retaining information associated with correct identification of the neural classes, while maintaining improvements in precision and overall F1-score. Moreover, the improvement over temporal features is strongly and negatively correlated with a subject’s temporal-only baseline accuracy (r=−0.88), indicating that participants with lower temporal-feature performance benefit the most from multi-domain feature integration. By comparison, the relationship between the improvement over cepstral features and baseline cepstral accuracy is weaker and positive (r=+0.41), indicating that subjects with higher cepstral performance tend to experience slightly larger additional gains from multi-domain fusion. Consequently, the modest improvement beyond cepstral features cannot be interpreted as primarily compensating for subjects who are poorly served by the cepstral representation alone. Instead, the consistently positive AMFF-versus-concatenation difference indicates that the adaptive weighting mechanism contributes additional discriminative value beyond simple feature aggregation, while the larger improvement relative to temporal features reflects the complementary information introduced by the cepstral domain.

**Figure 13:**
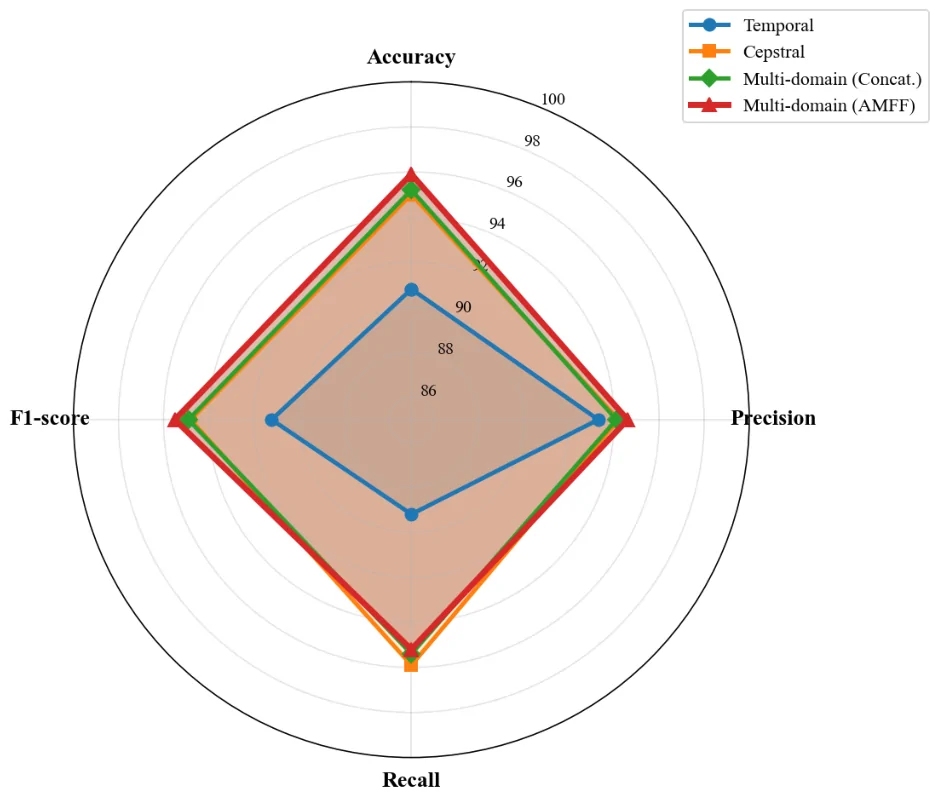
Radar plot comparing the average accuracy, precision, recall, and F1-score of the temporal, cepstral, multi-domain (concatenation), and multi-domain (AMFF) representations. The cepstral and both multi-domain representations overlap closely because their average scores fall within a narrow range (≈94–96%), and the temporal representation is distinctly lower on all four metrics. Exact per-metric values are reported in Table 2.

Figure 13 shows the result of analysis by viewing all four metrics simultaneously. The radar plot illustrates the distinction between direct concatenation and adaptive fusion of the temporal and cepstral domains. Both multi-domain representations substantially outperform the temporal representation across all four metrics, confirming the complementary contribution of cepstral information. However, the AMFF representation extends slightly beyond the simple concatenation representation for accuracy, precision, recall, and F1-score. This consistent outward shift across all four axes indicates that the improvement provided by AMFF is not restricted to a single evaluation criterion. In particular, the separation between AMFF and concatenation is most evident along the recall axis, consistent with the 0.50-percentage-point mean improvement observed in Table 2. These results suggest that adaptive fusion provides an additional refinement over direct concatenation while preserving the overall balanced performance obtained through multi-domain feature integration.

**Figure 14:**
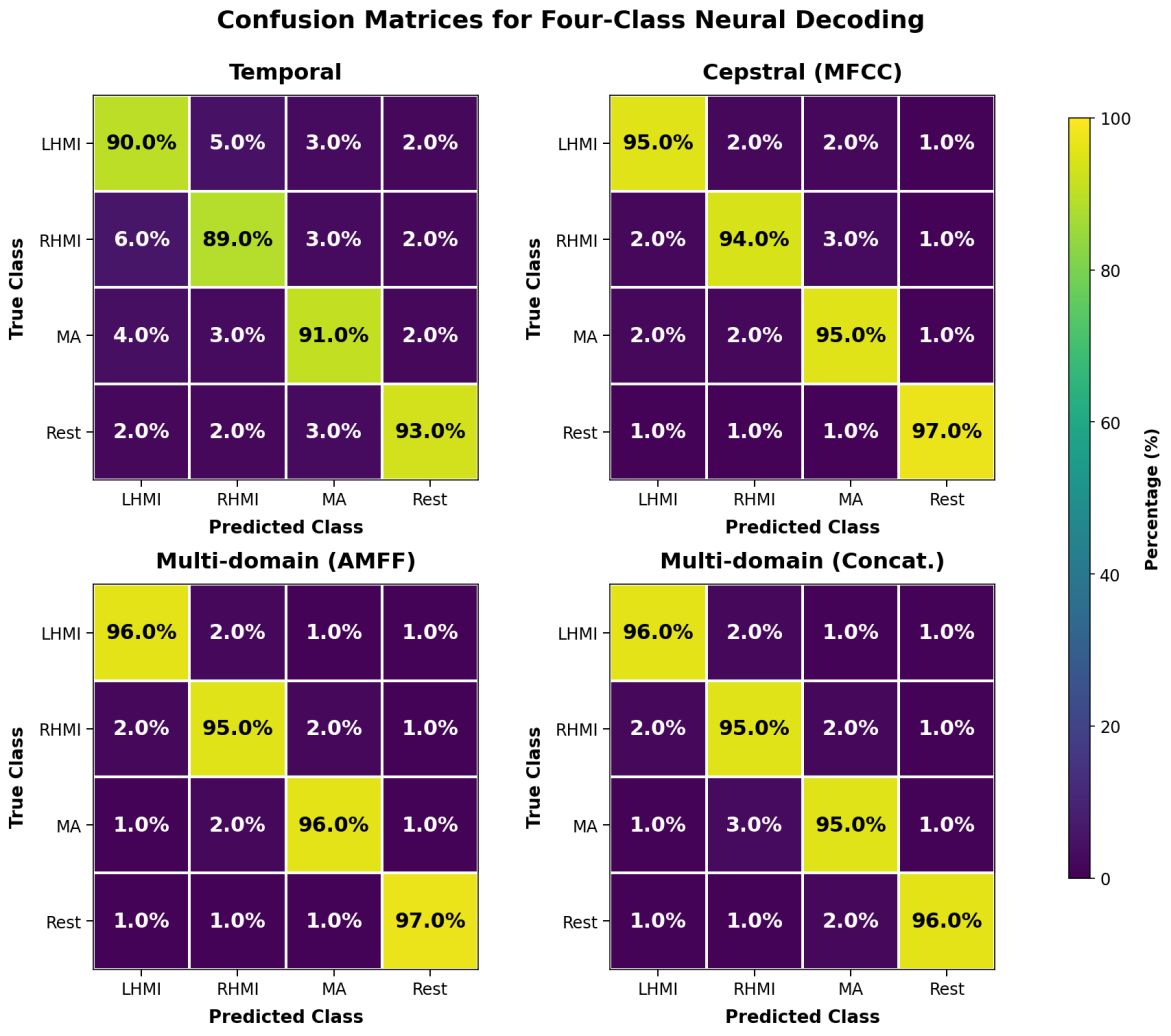
Normalized confusion matrices obtained from the temporal, cepstral (MFCC), and proposed multi-domain feature representations with AMFF and without AMFF (i.e., concatenation). Rows represent the true classes and columns represent the predicted classes. Values are reported as percentages for LHMI, RHMI, mental arithmetic (MA), and rest.

To further examine the class-wise classification behavior of the four feature representations, confusion matrices shown in Figure 14 were generated by comparing the pooled predicted labels with their corresponding ground-truth labels for the temporal, cepstral, concatenated, and AMFF feature sets. The confusion matrices provide a class-level analysis of the four-class neural decoding problem, comprising left-hand motor imagery (LHMI), right-hand motor imagery (RHMI), mental arithmetic (MA), and rest.

As shown in Figure 14, all four feature representations exhibit strong diagonal dominance, indicating that the majority of samples from each neural state were correctly classified. The temporal feature representation exhibits comparatively greater off-diagonal confusion, particularly between the two motor imagery classes and between motor imagery and mental arithmetic. This observation is consistent with the lower overall accuracy obtained using temporal descriptors alone.

The cepstral representation substantially reduces the off-diagonal errors and produces stronger diagonal classification rates across the four classes. This improvement is consistent with the higher average accuracy of 95.0% obtained using MFCC-based features compared with 90.7% for the temporal representation. Direct concatenation of temporal and cepstral descriptors further improves several diagonal entries, yielding classification rates of 96%, 95%, 95%, and 96% for LHMI, RHMI, MA, and rest, respectively. The proposed AMFF representation maintains or improves these diagonal rates, achieving 96%, 95%, 96%, and 97%, respectively.

The comparison between the two multi-domain representations is particularly informative. Direct concatenation already reduces the residual class confusion relative to the single-domain representations, confirming that temporal and cepstral descriptors provide complementary information. AMFF further increases the diagonal classification rate for mental arithmetic and rest while maintaining the same rates for LHMI and RHMI. Thus, although the numerical differences between concatenation and AMFF at the class level are modest, they are consistent with the subject-wise results, where AMFF provides higher accuracy, precision, recall, and F1-score across all 29 participants. Together, these findings indicate that the principal gain comes from integrating complementary temporal and cepstral information, while AMFF provides an additional refinement by adaptively weighting the combined representation rather than treating all concatenated features equally.

### 3.4. Statistical Significance Analysis

**Figure 15:**
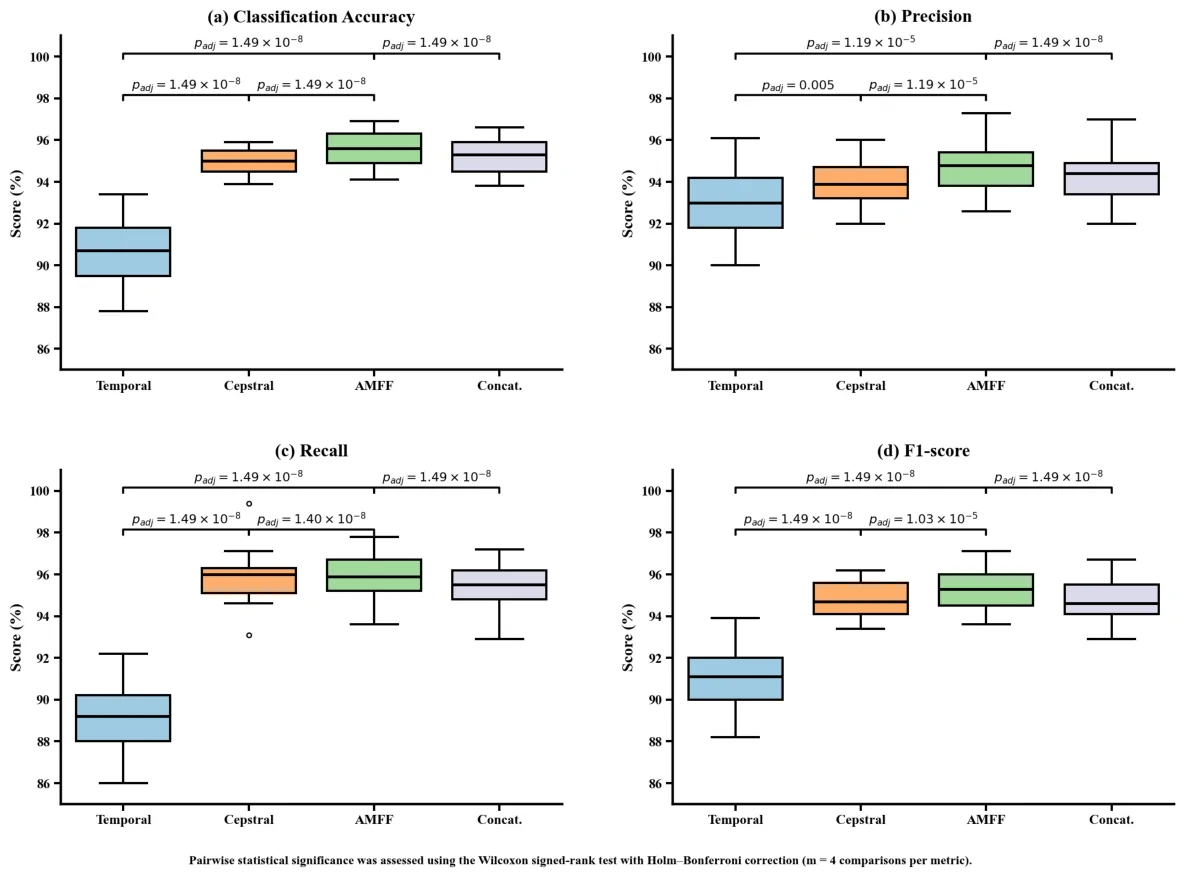
Distribution of subject-wise classification accuracy for the temporal, cepstral, multi-domain (concatenation), and multi-domain (AMFF) representations. Pairwise statistical significance obtained using the Wilcoxon signed-rank test with Holm–Bonferroni correction is indicated above the boxplots.

The descriptive results above show consistent, subject-wide improvements across the temporal, cepstral, and multi-domain feature representations. We therefore performed formal non-parametric statistical testing to determine whether the observed differences in subject-wise accuracy were statistically significant. Figure 15 presents the subject-wise accuracy distributions with the results of the pairwise statistical tests annotated above the boxplots. Because the four feature representations were evaluated on the same 29 subjects, a Friedman test was first performed to assess whether neural decoding accuracy differed significantly across the repeated-measures conditions.

The Friedman test revealed a statistically significant effect of feature representation on neural decoding accuracy ($\chi^{2}\left(3\right)=79.13$, $p=4.71\times 10^{-17}$), as summarized in Table 3. Kendall’s coefficient of concordance was W=0.910, indicating a high degree of concordance in the rank ordering of the four feature representations across subjects. This result demonstrates that the differences in accuracy were highly consistent across the 29 participants rather than being attributable to variation in only a small subset of subjects.

A statistically significant effect of feature representation was also observed for precision ($\chi^{2}\left(3\right)=57.17$, $p=2.37\times 10^{-12}$), with Kendall’s W=0.657. This indicates that the feature representations produced systematic differences in precision across subjects, although the lower concordance compared with accuracy suggests greater inter-subject variability in the relative ranking of the representations for this metric.

For recall, the Friedman test also indicated a statistically significant effect of feature representation ($\chi^{2}\left(3\right)=74.73$, $p=4.15\times 10^{-16}$), with a high Kendall’s *W* of 0.859. This demonstrates that the differences in recall were consistently expressed across the participants, indicating that the influence of feature representation on the ability to correctly identify positive trials was not driven by only a small number of subjects.

Similarly, F1-score showed a statistically significant effect of feature representation ($\chi^{2}\left(3\right)=76.18$, $p=2.02\times 10^{-16}$), with Kendall’s W=0.876. The high concordance indicates that the relative ordering of the feature representations in terms of their combined precision-recall performance was highly consistent across subjects. Collectively, these findings demonstrate that the effect of feature representation extends beyond classification accuracy and is consistently reflected in precision, recall, and F1-score across the 29 participants. Nevertheless, the Friedman test identifies only the presence of differences among the four representations; therefore, post hoc pairwise comparisons are required to determine which specific feature representations differ significantly for each evaluation metric.

### 3.5. Post Hoc Pairwise Comparisons

The Friedman test establishes that neural decoding accuracy differs significantly across the four feature representations but does not identify which specific pairs differ. Therefore, four theoretically motivated post hoc pairwise comparisons were performed using the exact Wilcoxon signed-rank test. Holm–Bonferroni correction was applied across the m=4 comparisons to control the family-wise error rate. Table 4 reports the signed-rank statistic *V*, the uncorrected *p*-value, the Holm-adjusted *p*-value, and the matched-pairs rank-biserial correlation *r* as a measure of paired effect direction and magnitude. The comparisons were selected to evaluate the contribution of cepstral features, the benefit of combining temporal and cepstral information using AMFF, and the additional effect of adaptive fusion relative to simple concatenation.

For accuracy, all four planned comparisons were statistically significant after Holm–Bonferroni correction ($p_{\mathrm{adj}}<1.50\times 10^{-8}$). The mean accuracy increased from 90.7% with temporal features to 95.0% with cepstral features, and further to 95.6% with AMFF. The corresponding AMFF improvement over cepstral features was 0.65 percentage points. AMFF also exceeded direct concatenation by 0.38 percentage points, with all 29 subjects showing higher accuracy using AMFF.

Precision showed a similar overall pattern. All four planned comparisons remained statistically significant after Holm–Bonferroni correction. Mean precision increased from 93.1% for temporal features to 93.9% for cepstral features and 94.6% for AMFF. The AMFF representation improved precision by 0.69 percentage points relative to the cepstral representation and by 0.41 percentage points relative to direct concatenation. The corresponding paired comparisons were significant, with adjusted *p*-values of $1.19\times 10^{-5}$ and $1.49\times 10^{-8}$, respectively.

For recall, the omnibus Friedman test was also highly significant. The transition from temporal to cepstral features produced a substantial increase in mean recall from 89.2% to 95.8%, and the temporal-versus-AMFF comparison was likewise statistically significant. AMFF produced a mean recall of 95.9%, compared with 95.8% for the cepstral representation and 95.4% for direct concatenation. The paired comparison between cepstral and AMFF recall remained significant after Holm–Bonferroni correction ($p_{\mathrm{adj}}=0.014$), but it was the weakest of the planned comparisons: 21 of the 29 subjects showed higher recall with AMFF, 6 showed lower recall, and 2 were tied, and the mean difference was only 0.08 percentage points. This difference is therefore statistically detectable but of negligible practical magnitude.

The F1-score results provide additional evidence for the contribution of the multi-domain representation. All four planned comparisons were statistically significant after Holm–Bonferroni correction. Mean F1-score increased from 91.1% with temporal features to 94.9% with cepstral features and 95.3% with AMFF. AMFF improved the mean F1-score by 0.43 percentage points relative to cepstral features and by 0.43 percentage points relative to direct concatenation. The corresponding paired comparisons yielded adjusted *p*-values below $1.1\times 10^{-5}$.

These analyses therefore provide statistical evidence for improvements beyond classification accuracy and demonstrate that the effects of feature representation extend to precision, recall, and F1-score. However, the results also indicate that the contribution of AMFF is metric-dependent. In particular, although AMFF differs significantly from the cepstral representation for all four metrics, the improvement in recall (0.08 percentage points) is negligible, whereas the improvements in accuracy, precision, and F1-score are larger (0.43–0.69 percentage points) but still modest. The relatively small absolute differences between AMFF and the strongest single-domain or concatenation baselines should also be considered when interpreting practical significance.

As a sensitivity analysis of the multiple-comparison procedure, the Holm–Bonferroni correction was additionally applied across the complete family of 16 metric×comparison tests, rather than separately within each evaluation metric. The resulting global Holm-adjusted *p*-values ranged from $2.61\times 10^{-8}$ to $1.44\times 10^{-2}$. Importantly, all 16 comparisons remained statistically significant at α=0.05 under this more conservative correction. Thus, the interpretation of the pairwise statistical results is unchanged by the broader correction scope. The effect-size estimates are unaffected by the correction because they are calculated from the underlying paired observations rather than from the adjusted *p*-values.

### 3.6. MFCC Parameter Sensitivity Analysis

Because MFCCs were originally developed for speech signals, their parameterization must be adapted carefully when applied to the substantially slower dynamics of fNIRS hemodynamic signals. To examine the robustness of the selected MFCC configuration, a parameter sensitivity analysis was conducted by systematically varying the frame length, frame overlap, and number of retained MFCC coefficients. Three frame lengths (64, 128, and 256 samples), three overlap ratios (25%, 50%, and 75%), and three MFCC dimensionalities (8, 10, and 12 coefficients) were evaluated, resulting in 27 parameter configurations. At the sampling frequency of 12.5 Hz, the evaluated frame lengths correspond to approximately 5.12, 10.24, and 20.48 s, respectively. All other preprocessing, feature extraction, classifier, training, and evaluation settings were kept unchanged to isolate the effect of the MFCC parameterization.

**Figure 16:**
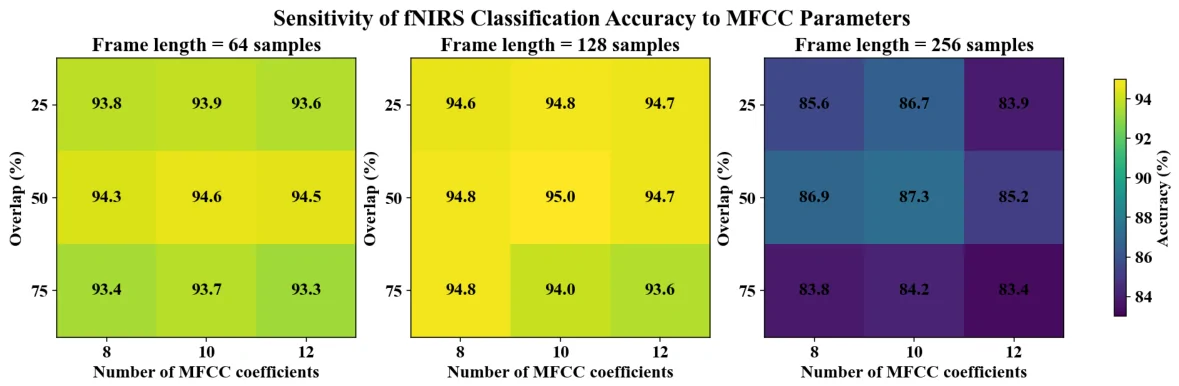
Sensitivity of fNIRS classification accuracy to MFCC parameterization. Each panel represents a different frame length, while rows and columns correspond to overlap ratio and number of retained MFCC coefficients, respectively. The values within the cells indicate classification accuracy (%).

The results are presented in Table 5 and Figure 16; the accuracies refer to the MFCC-only (cepstral) decoder. For the 64-sample frame length, classification accuracy ranged from 93.3% to 94.6%, with the highest accuracy obtained using 50% overlap and 10 MFCC coefficients. A similar but more stable pattern was observed for the 128-sample frame length, where accuracy ranged from 93.6% to 95.0%. The highest accuracy of 95.0% was obtained with a 128-sample frame, 50% overlap, and 10 MFCC coefficients, corresponding to the configuration adopted in the main experiments. In contrast, increasing the frame length to 256 samples resulted in a marked reduction in classification performance, with accuracies ranging from 83.4% to 87.3% across the evaluated overlap ratios and MFCC dimensionalities.

The observed degradation at 256 samples can be interpreted in the context of the temporal characteristics of the fNIRS signal. Although longer windows provide greater frequency resolution, they also reduce the temporal localization of the spectral representation. For slowly varying hemodynamic responses, an excessively long analysis window may therefore mix task-related and non-task-related temporal segments, reducing the discriminative utility of the resulting cepstral representation. Conversely, the 64- and 128-sample configurations provided a more favorable balance between spectral characterization and temporal localization.

The number of retained MFCC coefficients had a comparatively smaller effect than frame length. Across the 64- and 128-sample configurations, 8, 10, and 12 coefficients generally produced similar performance, although the 10-coefficient configuration yielded the highest accuracy for the selected 128-sample, 50%-overlap setting. Increasing the overlap from 50% to 75% did not provide a consistent improvement and, particularly for the 128-sample configuration, resulted in reduced performance when 10 and 12 coefficients were used. These results indicate that excessive overlap does not necessarily improve the discriminative representation of the hemodynamic signal.

The sensitivity analysis supports the use of a 128-sample frame, 50% overlap, and 10 MFCC coefficients as a suitable configuration for the present fNIRS decoding task. Importantly, the analysis also demonstrates that the selected configuration was not chosen solely on the basis of a single arbitrary parameter setting; rather, its performance was evaluated against systematically varied temporal and cepstral parameter configurations.

### 3.7. Analysis of Adaptive Fusion Weights

**Figure 17:**
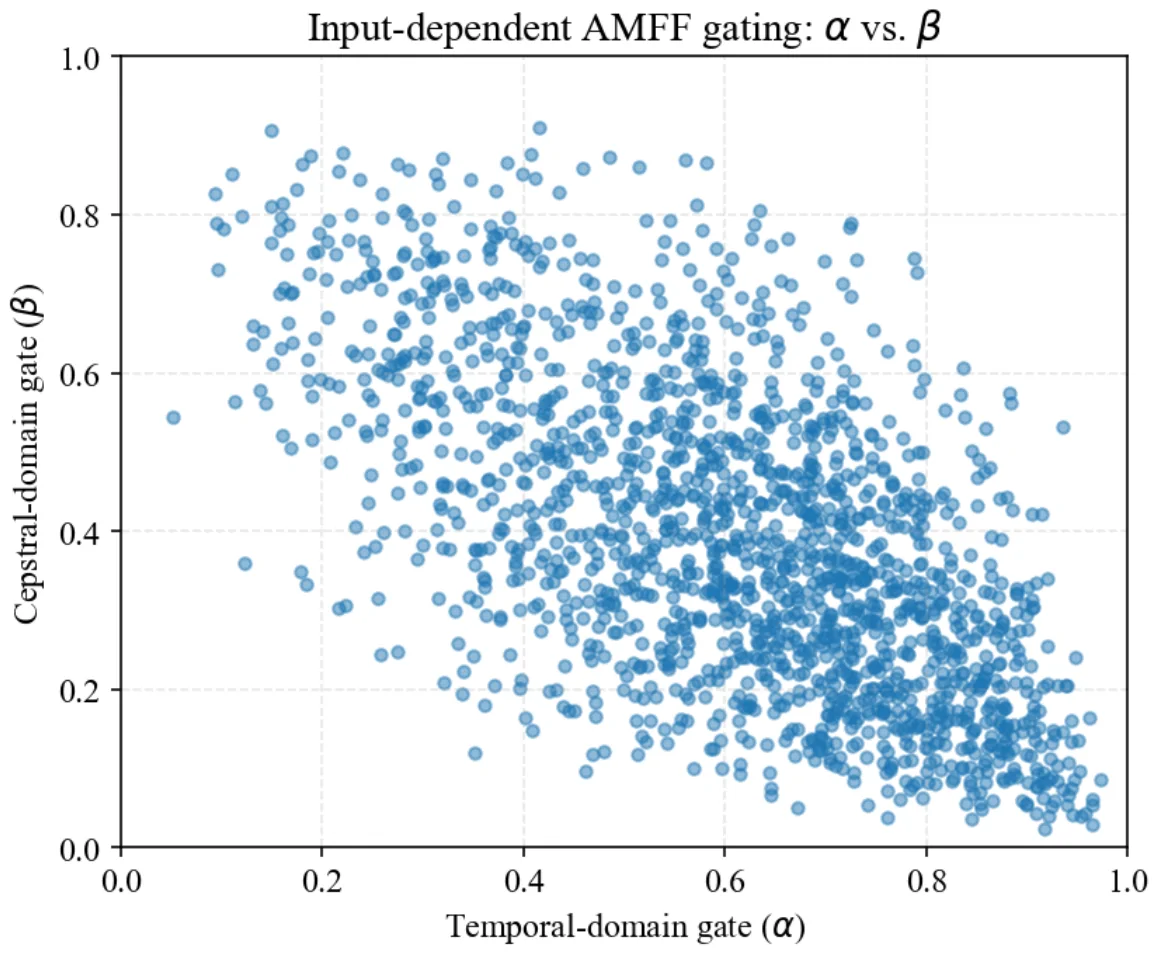
Relationship between the learned sample-dependent adaptive weights α and β across the held-out observations in the leave-one-subject-out evaluation. Each point represents one trial from the held-out subject of a LOSO fold. The temporal-domain gate α controls the contribution of the temporal hemodynamic descriptors, whereas the cepstral-domain gate β controls the contribution of the MFCC-based descriptors. The distribution of the points illustrates the input-dependent modulation of the relative contribution of the two feature domains.

To further investigate the behavior of the proposed adaptive fusion mechanism, the learned sample-dependent weights α and β were analyzed across the held-out observations from the LOSO evaluation. Recall that α and β are independently generated by sigmoid-activated weighting functions and therefore are not fixed parameters shared across all observations. Figure 17 illustrates the relationship between the temporal-domain gate (α) and the cepstral-domain gate (β) for the evaluated trials.

As shown in Figure 17, the learned weights exhibit a clear tendency toward an inverse relationship: observations receiving relatively greater weighting from the temporal feature domain tend to receive lower weighting from the cepstral domain, and vice versa. At the same time, the considerable dispersion of the observations indicates that the relative contribution assigned to the two domains is not fixed across trials. This behavior is consistent with the input-dependent formulation of AMFF, in which the contribution of each feature domain is dynamically modulated according to the corresponding input representation.

The learned weights exhibited a negative association between the two feature domains, with the temporal-domain gate and cepstral-domain gate showing a Pearson correlation of r=−0.62 (p<0.001). This indicates that observations assigned relatively greater weight to the temporal representation tended, on average, to receive lower weight from the cepstral representation. Nevertheless, the substantial dispersion around this trend demonstrates that the weighting is not governed by a fixed one-to-one relationship and varies across individual observations.

The observed variation in α and β provides additional evidence that AMFF does not simply apply a fixed weighting to the two feature domains. Instead, the model learns to adjust their relative contributions across individual observations. This provides a complementary interpretation of the performance comparison with static concatenation, where the temporal and cepstral features are combined without such input-dependent framework.

### 3.8. Feature Level Performance Analysis

The preceding analysis establishes that combining temporal and cepstral information improves performance, but treats the temporal side of the fusion as a single block of descriptors. To identify which individual temporal descriptors drive this benefit, MFCCs were combined with different subsets of three temporal hemodynamic descriptors at a time, and neural decoding accuracy was evaluated across all subjects for each combination. Table 6 summarizes the resulting mean accuracy and standard deviation.

Among all evaluated combinations, MFCCs combined with signal mean (SM), signal peak (SP), and kurtosis (KR) achieved the highest average accuracy of 92.1±2.1%, recovering approximately 29% of the accuracy gap between the temporal-only (90.7%) and multi-domain (95.6%) representations. Combinations involving variance and skewness generally resulted in lower performance and higher variability, indicating that these lower-order statistics are less discriminative than the mean, peak, and kurtosis descriptors when paired with MFCCs.

Two aspects of Table 6 require careful interpretation. First, every three-descriptor subset falls below both the standalone cepstral representation (95.0%) and the full multi-domain representation (95.6%) reported in Table 2. Truncating the temporal branch to three of the six descriptors therefore does not yield a cheaper deployable model: standalone MFCCs are both more accurate and lower-dimensional. This analysis should accordingly be read as a diagnostic ablation that isolates the relative contribution of individual temporal descriptors within the fused representation, and not as a recommendation for a reduced feature set. The most plausible explanation for the drop is that the adaptive gate is calibrated for the full six-descriptor temporal block, so removing half of that block leaves a temporal branch whose learned weighting no longer matches its reduced information content. Second, the subsets were evaluated under the same leave-one-subject-out protocol as Table 2, so the two tables are directly comparable.

**Figure 18:**
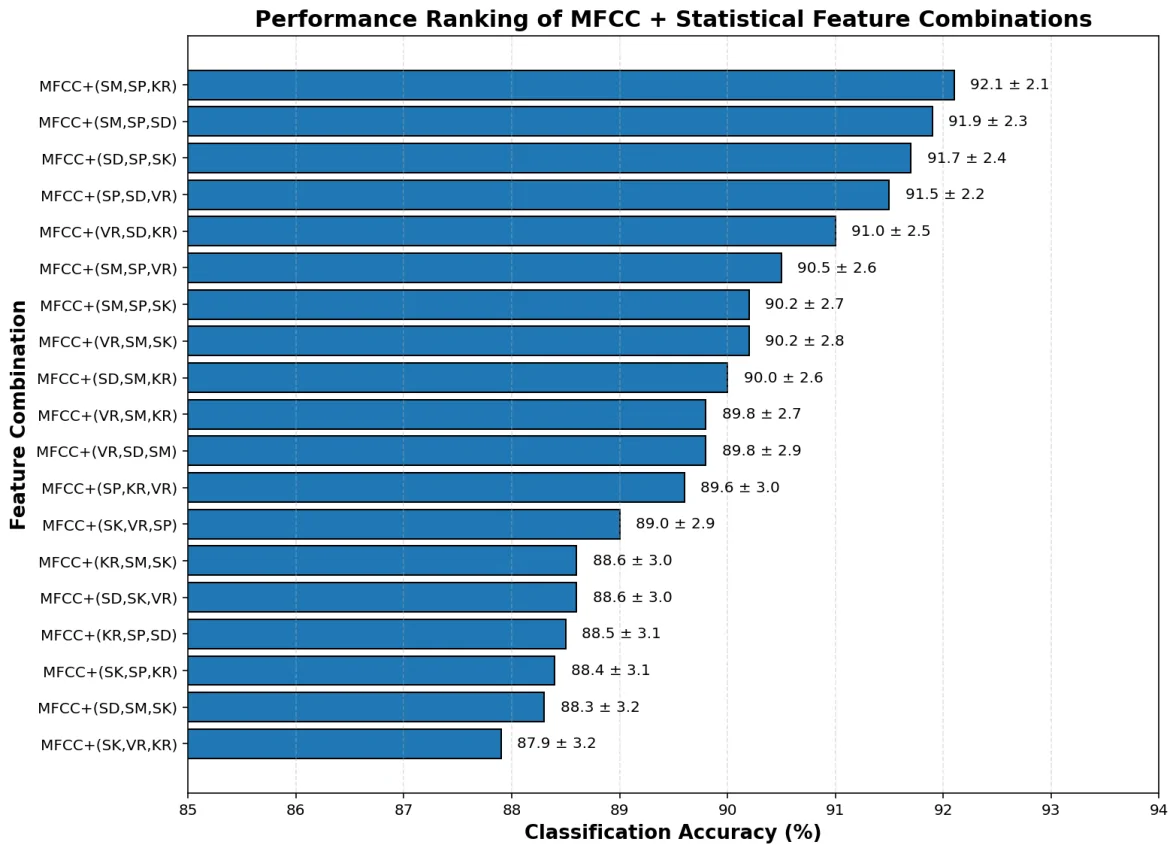
Ranking of MFCC combined with different three-feature statistical subsets according to the average neural decoding accuracy across 29 subjects. Error values denote the standard deviation.

Figure 18 presents the same combinations ranked visually, making the pattern in Table 6 easier to read at a glance. The four highest-ranked subsets all contain the signal peak, and the three lowest-ranked subsets all contain skewness, so the presence of the signal peak and the absence of skewness are the two clearest correlates of position in the ranking. The pattern is nonetheless not a strict one: the subset (SD, SP, SK) reaches 91.7% despite containing skewness, while (KR, SP, SD) falls to 88.5% despite containing both the peak and kurtosis, indicating that the descriptors interact rather than contribute additively. The overall spread between the best and worst subsets evaluated is roughly four accuracy points (92.1% to 87.9%).

**Figure 19:**
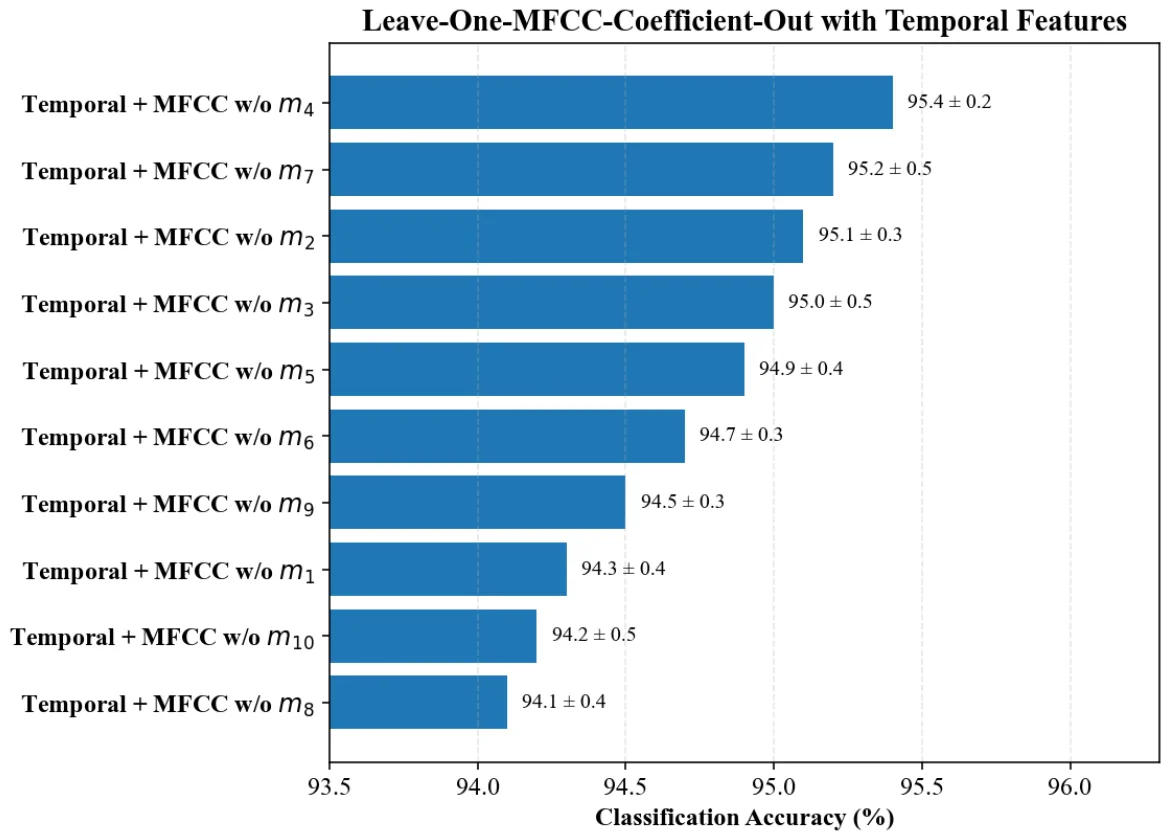
Leave-one-MFCC-coefficient-out ablation analysis.

To further examine the contribution of the cepstral representation, an individual MFCC coefficient ablation was performed by removing one coefficient at a time from the complete ten-coefficient MFCC representation while retaining the temporal descriptors and all other experimental settings unchanged. As shown in Table 7 and Figure 19, all leave-one-coefficient-out configurations resulted in lower accuracy than the complete representation, although the magnitude of the reduction varied across coefficients. Removing $m_{4}$ resulted in the smallest decrease, from 95.6% to 95.4% (0.2 percentage points), whereas removing $m_{8}$ produced the largest reduction, from 95.6% to 94.1% (1.5 percentage points). The remaining coefficients produced intermediate changes, ranging from 0.4 to 1.4 percentage points. These results indicate that the removal of individual MFCC coefficients affects decoding performance to different extents, suggesting that the coefficients make unequal marginal contributions within the complete cepstral representation. Nevertheless, the analysis should be interpreted as a leave-one-coefficient-out assessment of marginal contribution rather than as an independent measure of intrinsic feature importance, because the MFCC coefficients are jointly derived through the cepstral transformation and may contain correlated information.

The results in this section provide complementary evidence regarding the contribution of the temporal and cepstral feature representations. The main classification results show that cepstral descriptors substantially outperform the temporal-only representation, while combining the two domains through the proposed multi-domain fusion framework provides a further statistically significant improvement ($p_{\mathrm{adj}}<0.001$), with the improvement occurring for 28 of the 29 individual participants. The three-feature subset analysis further indicates that combinations containing the signal mean, signal peak, and kurtosis can achieve relatively higher performance among the evaluated temporal subsets, although the variability across combinations demonstrates that the descriptors interact and should not be interpreted as independently ranked according to intrinsic importance. Similarly, the leave-one-MFCC-coefficient-out analysis shows that removing individual cepstral coefficients produces different performance changes, with $m_{8}$ producing the largest decrease among the evaluated configurations. Taken together, these ablation analyses indicate that both temporal and cepstral information contribute non-uniformly to neural decoding performance, while also highlighting the importance of considering interactions among descriptors rather than interpreting individual features in isolation. These findings motivate the comparison with prior fNIRS-BCI work and discussion of practical implications presented next.

### 3.9. Chromophore Comparative Analysis: HbO, HbR, and Dual-Chromophore Fusion

To evaluate whether excluding HbR results in the loss of discriminative information, performance was benchmarked across HbO-only, HbR-only, and combined HbO+HbR feature representations under the same 29-subject LOSO evaluation protocol. For the combined HbO+HbR configuration, temporal and cepstral features were extracted from both chromophores and concatenated prior to adaptive fusion.

As summarized in Table 8, HbO-based feature extraction consistently outperformed HbR-based feature extraction across all evaluated representations. For the proposed AMFF framework, HbO achieved a mean accuracy of 95.6% compared with 91.8% for HbR. Dual-chromophore fusion (HbO+HbR) yielded a mean accuracy of 96.1% (precision 95.2%, recall 96.4%, F1-score 95.8%), providing a modest gain of 0.5 percentage points over HbO alone. These results confirm that while HbO captures the primary discriminative dynamics of the hemodynamic response, integrating HbR provides complementary chromophore-level information.

## 4. Discussion

This study investigated the effectiveness of multi-domain feature fusion for four-class fNIRS-based brain–computer interface (BCI) neural decoding using recordings from 29 participants. The classification task involved four distinct cognitive states: left-hand motor imagery (LHMI), right-hand motor imagery (RHMI), mental arithmetic (MA), and resting state. Unlike conventional binary BCI paradigms, four-class decoding requires the model to distinguish among multiple neural states with potentially overlapping hemodynamic characteristics. The low signal-to-noise ratio (SNR) of fNIRS, inter-subject variability, delayed hemodynamic responses, and non-stationary characteristics of the recorded signals further increase the difficulty of reliable classification [16,61,66]. These characteristics motivate the use of complementary feature representations capable of describing different aspects of the same underlying hemodynamic response.

The central contribution of this study is therefore the integration of heterogeneous feature domains rather than the use of a single feature representation. The proposed Adaptive Multi-Domain Feature Fusion (AMFF) framework combines conventional temporal hemodynamic descriptors with fNIRS-adapted Mel Frequency Cepstral Coefficients (MFCCs). Temporal descriptors characterize the amplitude, variability, distribution, and morphological properties of the hemodynamic response, whereas MFCCs provide a compact representation of its spectral characteristics. Accordingly, the two branches provide complementary descriptions of fNIRS activity. Rather than assuming that these representations are equally informative, AMFF introduces trainable weighting mechanisms to learn their relative contribution before forming the final feature representation. The resulting framework therefore addresses feature fusion as a representation-learning problem rather than as simple feature-vector concatenation.

The experimental results support the effectiveness of this multi-domain representation. The temporal feature domain achieved an average classification accuracy of 90.7%, whereas the MFCC feature domain achieved 95.0%. When the two domains were integrated using the proposed AMFF framework, the average accuracy increased to 95.6%, with precision, sensitivity, and F1-score of 94.6%, 95.9%, and 95.3%, respectively. The improvement obtained after integrating the two domains indicates that the information represented by the temporal and cepstral branches is not completely redundant. Instead, the cepstral representation provides information that complements the conventional temporal description, while the temporal descriptors retain amplitude and distributional characteristics that are not explicitly encoded by the MFCC representation. Statistical analysis using the Friedman test followed by Wilcoxon signed rank tests further supports significant differences among the evaluated feature representations. Collectively, these findings demonstrate the value of multi-domain representation for four-class fNIRS neural decoding.

### 4.1. Comparison with Existing fNIRS-BCI Studies

The present findings are consistent with the increasing emphasis on richer feature representations and information fusion in fNIRS-based BCI research. However, direct numerical comparison with previous studies should be interpreted cautiously because differences in datasets, participants, cognitive paradigms, preprocessing procedures, feature representations, classifiers, and validation protocols can substantially affect reported performance. The comparisons presented here therefore provide contextual evidence regarding the development of fNIRS decoding rather than controlled head-to-head benchmarks.

Earlier fNIRS studies have predominantly relied on handcrafted time-domain or statistical features. Naseer et al. demonstrated the effectiveness of optimized statistical features for fNIRS-based motor imagery classification, establishing the usefulness of temporal descriptors for discriminating task-related hemodynamic responses [19,20]. Such features remain attractive because they are computationally inexpensive and relatively interpretable. However, their representation is primarily based on amplitude and distributional characteristics and therefore provides only a partial description of the signal. The temporal-feature accuracy of 90.7% obtained in the present study confirms that these descriptors remain informative, while the higher performance obtained using MFCCs suggests that additional spectral information can provide substantial discriminative value.

Deep learning has subsequently enabled automatic representation learning from fNIRS signals. Asgher et al. investigated CNN and LSTM models for four-level mental workload classification and reported average accuracies of 87.45% and 89.31%, respectively [67]. Although their four-class task is different from the LHMI, RHMI, MA, and resting-state paradigm considered here, the study demonstrates the feasibility of multiclass fNIRS decoding. Other studies have similarly explored convolutional, recurrent, graph-based, and attention-based architectures [22,23,24,25]. These approaches provide powerful representation-learning capabilities, but often require substantially more complex architectures and larger amounts of training data. The results obtained in the present study suggest that improving the feature representation through complementary domain fusion can provide an alternative route to improving decoding performance while retaining a lightweight MLP classifier.

An important distinction of the present study is the explicit incorporation of cepstral information. Ghaffar et al. introduced MFCCs for fNIRS-based neural decoding and demonstrated that cepstral coefficients can provide useful discriminative information [34]. The standalone MFCC accuracy of 95.0% obtained in the present study provides further evidence that the spectral characteristics of fNIRS signals contain information relevant to cognitive-state discrimination. However, the objective of the present study is not simply to demonstrate the usefulness of MFCCs. Rather, MFCCs are treated as one feature domain within a broader multi-domain representation. The increase from 95.0% for MFCCs alone to 95.6% after integration with temporal descriptors demonstrates that the strongest representation is obtained by exploiting complementary information from both domains.

Recent research has increasingly recognized the value of feature fusion and multimodal representation learning. Khani et al. demonstrated the benefit of fusing multiple time-frequency representations derived from fNIRS signals, showing that different representations of the same underlying haemodynamic activity can provide complementary information [14]. In parallel, several EEG-fNIRS studies have investigated multi-domain, feature-level, decision-level, and attention-based fusion strategies [13,35,37,38,40,41,42]. More recent approaches have further introduced correlation-aware, multi-granularity, and cross-attention-based fusion mechanisms [44,45,47]. These studies collectively indicate a shift from single-domain representation toward learned integration of complementary information.

The present work extends this direction in a different manner by considering heterogeneous feature domains within a single fNIRS modality. Unlike EEG-fNIRS fusion, which combines information from two different sensing modalities, the proposed framework integrates two representations derived from the same hemodynamic signal. This avoids the additional heterogeneity associated with different sensing modalities while still allowing complementary temporal and spectral information to be exploited. Furthermore, the proposed approach uses a lightweight adaptive weighting mechanism rather than a large cross-modal attention architecture. This provides a computationally simpler framework for investigating whether different fNIRS feature domains should contribute equally to the final neural representation.

### 4.2. Contribution of Multi-Domain Feature Fusion

The main finding of this study is that combining temporal and cepstral representations improves neural decoding performance compared with the temporal representation alone. Temporal descriptors summarize characteristics such as mean, variance, standard deviation, skewness, kurtosis, and peak, whereas MFCCs provide a compact representation of the spectral characteristics of the hemodynamic signal. The two representations therefore describe complementary characteristics of the same fNIRS response.

The performance progression across the four representations provides a clearer separation between the contribution of feature combination and the contribution of adaptive fusion. Mean accuracy increased from 90.7% for temporal features to 95.0% for MFCCs. Direct concatenation of the temporal and cepstral features further increased accuracy to 95.2%, while the proposed AMFF representation achieved 95.6%. Therefore, most of the improvement over the temporal representation is associated with the cepstral representation, whereas the temporal features provide a smaller additional contribution when combined with MFCCs. Importantly, the comparison between static concatenation and AMFF isolates the additional contribution of adaptive fusion: AMFF improved accuracy by 0.4 percentage points over direct concatenation. Thus, the results indicate that the primary performance gain arises from incorporating complementary cepstral information, while adaptive fusion provides a further but relatively modest refinement.

The advantage of AMFF over direct concatenation was also consistent across the four evaluation metrics. Relative to concatenation, AMFF improved accuracy, precision, recall, and F1-score by approximately 0.38, 0.41, 0.50, and 0.43 percentage points, respectively. Moreover, AMFF achieved higher accuracy than direct concatenation for all 29 participants. The consistency of these paired improvements resulted in a statistically significant difference ($p_{\mathrm{adj}}<0.001$). However, the relatively small absolute differences indicate that the statistical significance primarily reflects the consistency of the improvement across subjects rather than a large practical increase in mean accuracy. Accordingly, the contribution of AMFF should be interpreted as a consistent incremental benefit over static feature combination rather than as the primary source of the overall decoding improvement.

The three-feature temporal ablation provides additional information about the interaction between the temporal and cepstral domains. Among the evaluated three-descriptor subsets combined with MFCCs, the combination of signal mean (SM), signal peak (SP), and kurtosis (KR) achieved the highest average accuracy of 92.1%. However, all evaluated three-descriptor subsets remained below both the standalone MFCC representation and the complete multi-domain representations. These results therefore do not support replacing the complete temporal representation with a reduced three-descriptor subset. Instead, the analysis serves as a diagnostic assessment of how different temporal descriptor combinations behave when paired with cepstral information. The variation among subsets further indicates that the temporal descriptors interact with one another and with the cepstral representation rather than contributing independently.

The individual MFCC ablation provides a complementary analysis of the cepstral branch. Removing each MFCC coefficient individually produced different changes in decoding accuracy. Removal of $m_{4}$ resulted in the smallest reduction, from 95.6% to 95.4%, whereas removal of $m_{8}$ produced the largest reduction, to 94.1%. The remaining coefficients produced intermediate changes. These findings indicate that individual coefficients make different marginal contributions within the complete cepstral representation. Nevertheless, these results should not be interpreted as direct measures of intrinsic feature importance because the MFCC coefficients are jointly derived through the cepstral transformation and may contain correlated information.

In short, the experiments distinguish three related effects. First, cepstral features provide a substantial improvement over the temporal-only representation. Second, combining temporal and cepstral information through either direct concatenation or AMFF provides a further improvement over the temporal representation, demonstrating the complementary nature of the two domains. Third, AMFF provides a small but consistent improvement over static concatenation, indicating an additional contribution from adaptive weighting. The adaptive weights further provide an input-dependent mechanism for modulating the relative contribution of the temporal and cepstral branches, as examined separately in the adaptive fusion weight analysis. Together, these results support AMFF as a compact adaptive fusion mechanism, while avoiding the stronger claim that adaptive weighting alone is responsible for the overall improvement in decoding performance.

### 4.3. Implications for fNIRS-BCI Applications

The findings have practical implications for the development of multi-class fNIRS-based BCIs. Increasing the number of command classes generally increases the difficulty of reliable classification because the decision boundaries between cognitive states become less distinct. Consequently, improving the richness of the input representation is an important complement to increasing classifier complexity.

The proposed framework demonstrates that this improvement can be achieved through multi-domain feature integration while retaining a lightweight MLP decoder. This is particularly relevant for portable and wearable fNIRS-BCI applications, where computational resources, model size, and processing latency may constrain the use of large deep architectures. The use of interpretable temporal descriptors together with compact MFCC representations also provides a transparent representation-level explanation of how complementary information is introduced into the classifier.

From a broader perspective, the results suggest that feature fusion should not necessarily be restricted to multimodal systems. The same fNIRS recording can support multiple complementary representations, including temporal, spectral, time-frequency, entropy-based, connectivity-based, and spatial features. Consequently, adaptive multi-domain fusion provides a potentially general strategy for improving fNIRS neural decoding without requiring additional sensing modalities.

### 4.4. Limitations and Future Directions

Several limitations should be considered when interpreting the present findings. First, the experiments were conducted using recordings from 29 participants, with 120 trials per participant (30 trials for each of the four experimental classes, totaling 3480 trial samples). Although this provides a substantial total sample size across the cohort, the number of trials available per class within an individual participant (30 trials) remains modest. To reduce the potential influence of within-subject dependence and prevent information leakage, the present study used a subject-independent leave-one-subject-out (LOSO) protocol, with participant identity serving as the unit of partitioning. Performance was subsequently summarized across the 29 held-out participants using mean and standard deviation, while subject-wise results were also reported to expose inter-subject variability. Nevertheless, the limited number of participants and trials per class remains a limitation of the present study. Validation using larger and more diverse cohorts, with a greater number of repeated trials per class, is required to obtain more precise estimates of performance and to establish the generalizability of the proposed multi-domain fusion strategy.

Second, the experiments were conducted under controlled laboratory conditions. Real-world fNIRS-BCI systems are likely to encounter motion artifacts, physiological fluctuations, user fatigue, changes in cognitive state, and inter-session variability. Future studies should therefore investigate the stability of the learned multi-domain representation across recording sessions and more naturalistic conditions.

Third, while our comparative experiments in Section 3.9 evaluate HbO, HbR, and combined HbO+HbR feature sets—confirming that dual-chromophore fusion reaches 96.1% accuracy—the primary AMFF architecture was kept centered on HbO to isolate temporal–cepstral feature-domain fusion. Future work will explore integrated multi-level fusion architectures that simultaneously learn adaptive weights across both feature domains (temporal vs. cepstral) and chromophores (HbO vs. HbR).

Fourth, although the static concatenation baseline shows that adaptive weighting adds a consistent improvement, the margin is small (0.2–0.7 percentage points per subject). Comparisons with fixed-weight fusion and with other gating or attention-based fusion schemes, and a direct comparison of the Mel filter bank with an otherwise identical linear filter bank, were not performed and remain future work.

Finally, the adaptive fusion concept can potentially be extended beyond the two feature domains considered here. Future work could investigate multi-branch fusion architectures capable of dynamically weighting temporal, spectral, time-frequency, spatial, and connectivity representations. The framework could also be extended to multimodal EEG-fNIRS systems, where adaptive fusion could simultaneously exploit within-modality feature complementarity and cross-modality neural information. Such extensions would provide a broader evaluation of adaptive multi-domain representation learning for robust neural decoding.

## 5. Conclusions

This study presented an Adaptive Multi-Domain Feature Fusion (AMFF) framework that integrates temporal hemodynamic descriptors and fNIRS-adapted MFCCs through sample-dependent weights for four-class fNIRS-BCI decoding. Under subject-independent leave-one-subject-out validation on 29 participants, AMFF reached a mean accuracy of 95.6% (precision 94.6%, recall 95.9%, F1-score 95.3%), compared with 90.7% for temporal descriptors, 95.0% for MFCCs, and 95.2% for static concatenation of the same features with the same classifier. These results separate two effects. Most of the improvement over temporal descriptors comes from adding cepstral information, while adaptive weighting provides a further improvement over static concatenation that is small (0.2–0.7 percentage points) but consistent across all 29 participants and all four metrics. AMFF is therefore best regarded as a lightweight refinement of multi-domain fusion rather than the main source of the decoding gain. The sensitivity and ablation analyses showed that frame length is the most influential MFCC parameter and that individual temporal descriptors and MFCC coefficients contribute unequally and interactively. Future work will evaluate HbR and joint HbO-HbR representations, fixed-weight and attention-based fusion alternatives, additional feature domains, cross-session and multi-center data, and extension to hybrid EEG-fNIRS systems.

## Figures and Tables

**Table 1:** Comparison of representative feature-fusion approaches in fNIRS and hybrid EEG-fNIRS BCI systems.

Ref.	Modality	Feature/Representation	Fusion Strategy	Main Contribution	Limitation/Gap
[13]	fNIRS/ EEG-fNIRS	Temporal statistical features + PSD features of HbO/HbR	Multi-domain feature hybridization followed by progressive learning	Demonstrated that combining temporal and PSD features within fNIRS improved MI and MA classification compared with individual feature domains; also investigated EEG-fNIRS multimodal fusion	Uses conventional PSD-based spectral features and progressive learning; does not investigate temporal–cepstral fusion or adaptive weighting between heterogeneous fNIRS feature domains
[14]	fNIRS	2D-DOST time-frequency representations of HbO, HbR, and their combinations	Deep feature fusion	Demonstrated that complementary time-frequency representations derived from a single fNIRS modality can be fused to improve subject-independent motor-execution classification	Focuses on multiple time-frequency representations and deep feature fusion rather than the fusion of handcrafted temporal statistical and MFCC-based cepstral domains
[49]	fNIRS	Frequency-domain, high-density, Oxy/deOxy Hb topographies	Layered topographical maps of hemoglobin changes + 3D-CNN spatio-temporal features	Simultaneous spatial–temporal feature extraction within a single 3D-CNN; probe-design-driven spatial–temporal feature extraction and improved subject-independent classification compared with a temporal CNN	Small cohort (7 subjects); F1 remained below real-time BCI thresholds
[37]	EEG-fNIRS	Selective channel representations and spectrogram images	Representation/feature integration	Combined complementary channel and spectrogram information for multimodal classification	Requires two sensing modalities and does not investigate single-modality fNIRS feature-domain fusion
[38]	EEG-fNIRS	Deep representations from separate EEG and fNIRS networks	Decision-level fusion	Demonstrated the effectiveness of combining modality-specific decisions for cognitive-task classification	Fusion occurs at the decision level and does not adaptively integrate heterogeneous fNIRS feature vectors
[41]	EEG-fNIRS	Learned multimodal representations	Cross-modality attentional fusion	Used attention to selectively exploit complementary EEG and fNIRS information	Designed for multimodal fusion rather than temporal and cepstral feature fusion within fNIRS
[40]	EEG-fNIRS	Learned EEG and fNIRS features	Feature- and decision-level fusion with attention	Demonstrated progressive integration of multimodal information using attention-based fusion	Relies on EEG-fNIRS multimodality and deep feature learning rather than explicit fNIRS temporal/cepstral fusion
[42]	EEG-fNIRS	Spatial–temporal representations	Spatial–temporal convolution and dual attention	Improved multimodal representation through spatial–temporal modeling and attention	High-complexity multimodal architecture; does not examine lightweight fNIRS feature-domain fusion
[39]	EEG-fNIRS	Modality-specific deep representations	Two-level evidence/decision fusion	Integrated uncertainty modeling and evidence theory into multimodal decision fusion	Operates at decision level and targets multimodal uncertainty rather than fNIRS feature-domain complementarity
[43]	EEG-fNIRS	Temporal and spatial representations	Temporal–spatial fusion	Explicitly modeled complementary temporal and spatial information in hybrid BCI	Multimodal and architecture-heavy; does not address temporal versus cepstral fNIRS descriptors
[50]	fNIRS	Deep features from 1D-CNN, LSTM, and Bi-LSTM + FFT-enhanced frequency-domain features	Stacking ensemble of deep-feature classifiers and FFT-transformed deep features	Feature stacking and frequency-domain enhancement increased accuracy to 90.11% compared with 79–85% for conventional CNN, LSTM, and Bi-LSTM models	Intra-subject evaluation only; two-class hand-gripping task; no cross-subject or online validation
[51]	fNIRS	Multi-scale fused features extracted from fNIRS time series	Multi-scale feature fusion + improved Transformer with adaptive dynamically gated attention weights	Selective enhancement of key features and noise suppression; achieved up to 77.97% accuracy, with ablation results confirming that fused features outperformed single-feature inputs	Conference-scale validation; 5 × 5 and leave-one-out cross-validation only; no cross-dataset generalization test
[48]	fNIRS	Gramian angular difference field (GADF) image features + hemodynamic-weighted time-series features	Two parallel feature modules fused with learnable normalization weights (VisiTempNet)	Combined image-based GADF and time-series features for fNIRS classification; achieved 76.65 ± 2.43% on the fNIRS2MW dataset and outperformed the evaluated baselines	Two-class mental-workload dataset only; intra-subject accuracy remained near the BCI threshold; conference-stage validation
[46]	EEG-fNIRS	Common and modality-specific spatiotemporal representations	Hybrid early/late fusion with attention	Separates common and modality-specific information while performing multimodal fusion	Focuses on EEG-fNIRS representation disentanglement rather than single-modality fNIRS feature fusion
[44]	EEG-fNIRS	Intra- and inter-modality representations	Correlation-based multimodal fusion	Models relationships within and between EEG and fNIRS representations	Requires multimodal data and targets inter-modality relationships
[45]	EEG-fNIRS	Multi-granularity representations	Multi-granularity fusion with sparse routing	Learns selective fusion across different representation granularities	Designed for complex multimodal deep learning rather than interpretable handcrafted feature-domain fusion
[47]	EEG-fNIRS	Hierarchical spatiotemporal features	Adaptive temporal alignment and symmetric cross-attention fusion	Performs adaptive alignment and deep cross-modal feature interaction	Addresses EEG-fNIRS temporal heterogeneity; additional alignment complexity is unnecessary for single-modality fNIRS
**Proposed AMFF**	fNIRS	Temporal statistical descriptors + MFCC-based cepstral descriptors	Adaptive feature-level domain weighting	Learns the relative contribution of temporal and cepstral information before neural decoding	Addresses the underexplored fusion of heterogeneous temporal and cepstral feature domains within a single fNIRS modality

**Table 2:** Subject-wise neural decoding performance (%) for four feature sets in the fNIRS-BCI system.

Subjects	Features															
	Temporal (%)				Cepstral (%)				Multi-Domain (AMFF) (%)				Multi-Domain (Concat.) (%)			
	Acc	Prec	Rec	F1	Acc	Prec	Rec	F1	Acc	Prec	Rec	F1	Acc	Prec	Rec	F1
1	92.7	96.1	87.3	91.4	95.9	95.6	96.0	95.7	96.4	97.3	93.6	95.4	95.8	97.0	92.9	95.0
2	89.5	94.2	88.9	91.4	94.8	92.0	95.0	93.4	94.7	92.6	96.5	94.5	94.3	92.0	96.2	94.3
3	93.4	95.7	92.2	93.9	95.5	93.2	99.4	96.2	96.9	94.9	97.8	97.1	96.6	94.5	97.2	96.7
4	90.1	90.0	91.8	90.8	95.0	96.0	93.1	94.5	95.5	93.1	94.2	93.6	94.9	92.5	93.9	92.9
5	87.8	90.7	86.0	88.2	93.9	92.3	96.0	94.1	94.1	95.5	93.9	94.6	93.8	95.0	93.2	94.1
6	91.3	93.6	88.7	91.1	94.6	93.8	95.1	94.4	95.6	94.8	95.9	95.3	95.4	94.4	95.2	94.6
7	88.9	92.1	87.4	89.7	95.2	94.5	96.2	95.3	96.1	95.2	96.7	96.0	95.9	94.9	96.1	95.8
8	92.1	94.8	90.3	92.5	94.1	92.9	95.0	93.9	94.9	93.6	95.3	94.4	94.5	93.4	94.8	93.7
9	89.7	91.9	88.1	90.0	95.6	94.7	96.8	95.7	96.3	95.4	96.8	96.1	96.1	94.9	96.2	95.5
10	90.8	93.0	89.4	91.2	94.7	93.6	95.2	94.4	95.2	94.1	95.6	94.8	94.9	93.5	95.2	94.4
11	91.6	94.1	89.9	91.9	95.3	94.9	96.0	95.4	96.0	95.1	96.4	95.7	95.7	94.8	95.9	95.2
12	88.6	91.5	87.2	89.3	94.0	93.1	94.6	93.8	94.8	93.7	95.1	94.4	94.5	93.5	94.6	93.8
13	92.9	95.2	91.0	93.0	95.7	94.2	97.1	95.6	96.5	95.6	97.0	96.3	96.2	95.3	96.6	95.7
14	90.4	92.8	89.6	91.1	94.9	93.8	95.5	94.6	95.3	94.2	95.8	95.0	94.9	93.5	95.6	94.4
15	89.2	91.7	87.9	89.8	94.3	92.7	95.1	93.9	94.6	93.5	95.0	94.3	94.1	93.3	94.8	94.1
16	91.0	93.5	89.2	91.3	95.1	94.3	96.0	95.1	95.8	94.9	96.2	95.5	95.4	94.7	95.6	95.1
17	90.2	92.1	88.8	90.9	94.6	93.5	95.3	94.4	95.1	94.0	95.6	94.8	94.9	93.4	95.3	94.3
18	92.4	94.6	91.1	92.8	95.8	95.0	97.0	96.0	96.6	95.8	97.2	96.5	96.1	95.5	96.5	96.1
19	89.9	91.4	88.5	90.1	94.4	93.2	95.0	94.1	94.9	93.8	95.2	94.5	94.7	93.4	94.7	94.3
20	90.7	92.9	89.3	91.0	95.0	94.1	96.2	95.1	95.7	94.6	96.0	95.3	95.5	94.3	95.5	95.1
21	91.8	94.0	90.2	92.0	95.6	94.7	96.9	95.8	96.4	95.5	96.9	96.2	96.2	94.9	96.2	96.0
22	88.7	90.9	87.1	89.0	94.1	93.0	94.8	93.9	94.7	93.6	95.0	94.3	94.4	93.3	94.3	94.1
23	92.0	94.5	90.7	92.4	95.4	94.6	96.3	95.4	96.2	95.3	96.7	96.0	95.6	94.8	96.1	95.5
24	89.4	91.8	88.0	89.8	94.5	93.4	95.2	94.3	95.0	93.9	95.4	94.6	94.4	93.3	95.2	94.0
25	90.9	93.1	89.5	91.2	95.2	94.3	96.1	95.2	95.9	94.8	96.3	95.5	95.3	94.6	95.8	95.0
26	91.5	94.2	90.0	92.0	95.7	94.9	96.8	95.8	96.5	95.7	97.1	96.4	96.0	95.4	96.6	96.0
27	88.8	91.0	87.6	89.2	94.2	93.1	94.9	94.0	94.8	93.7	95.2	94.4	94.5	93.2	94.7	94.1
28	92.6	95.0	91.5	93.2	95.9	95.2	97.1	96.1	96.8	96.0	97.4	96.7	96.1	95.5	96.7	96.3
29	90.0	92.3	88.9	90.6	94.8	93.9	95.6	94.7	95.4	94.3	95.8	95.0	95.1	93.7	95.4	94.6
Average	90.7	93.1	89.2	91.1	95.0	93.9	95.8	94.9	95.6	94.6	95.9	95.3	95.2	94.2	95.4	94.9

**Table 3:** Friedman test results for all four performance metrics across the four feature representations.

Metric	$\chi^{2}\left(3\right)$	*p*-Value	Kendall’s *W*	*N*
Accuracy	79.13	$4.71\times 10^{-17}$	0.910	29
Precision	57.17	$2.37\times 10^{-12}$	0.657	29
Recall	74.73	$4.15\times 10^{-16}$	0.859	29
F1-score	76.18	$2.02\times 10^{-16}$	0.876	29

**Table 4:** Post hoc pairwise comparisons of subject-wise performance across feature representations using the exact Wilcoxon signed-rank test. Adjusted *p*-values were obtained using the Holm–Bonferroni correction within each evaluation metric. *r* denotes the matched-pairs rank-biserial correlation. ^†^ Two zero differences and tied ranks were present; this *p*-value uses the normal approximation with zero differences discarded.

Metric	Comparison	*V*	Raw *p*	Adjusted *p*	*r*	Significance
Accuracy	Temporal vs. Cepstral	0	$3.73\times 10^{-9}$	$1.49\times 10^{-8}$	+1.000	Significant
	Cepstral vs. Multi-domain (AMFF)	1	$7.45\times 10^{-9}$	$1.49\times 10^{-8}$	+0.995	Significant
	Temporal vs. Multi-domain (AMFF)	0	$3.73\times 10^{-9}$	$1.49\times 10^{-8}$	+1.000	Significant
	Multi-domain (Concat.) vs. Multi-domain (AMFF)	0	$3.73\times 10^{-9}$	$1.49\times 10^{-8}$	+1.000	Significant
Precision	Temporal vs. Cepstral	91	$5.16\times 10^{-3}$	$5.16\times 10^{-3}$	+0.582	Significant
	Cepstral vs. Multi-domain (AMFF)	28	$5.52\times 10^{-6}$	$1.19\times 10^{-5}$	+0.871	Significant
	Temporal vs. Multi-domain (AMFF)	26	$3.98\times 10^{-6}$	$1.19\times 10^{-5}$	+0.880	Significant
	Multi-domain (Concat.) vs. Multi-domain (AMFF)	0	$3.73\times 10^{-9}$	$1.49\times 10^{-8}$	+1.000	Significant
Recall	Temporal vs. Cepstral	0	$3.73\times 10^{-9}$	$1.49\times 10^{-8}$	+1.000	Significant
	Cepstral vs. Multi-domain (AMFF)	87.5	$1.44\times 10^{-2}$	$1.44\times 10^{-2}$	+0.537	Significant ^†^
	Temporal vs. Multi-domain (AMFF)	0	$3.73\times 10^{-9}$	$1.49\times 10^{-8}$	+1.000	Significant
	Multi-domain (Concat.) vs. Multi-domain (AMFF)	0	$3.73\times 10^{-9}$	$1.49\times 10^{-8}$	+1.000	Significant
F1-score	Temporal vs. Cepstral	0	$3.73\times 10^{-9}$	$1.49\times 10^{-8}$	+1.000	Significant
	Cepstral vs. Multi-domain (AMFF)	32	$1.03\times 10^{-5}$	$1.03\times 10^{-5}$	+0.853	Significant
	Temporal vs. Multi-domain (AMFF)	0	$3.73\times 10^{-9}$	$1.49\times 10^{-8}$	+1.000	Significant
	Multi-domain (Concat.) vs. Multi-domain (AMFF)	0	$3.73\times 10^{-9}$	$1.49\times 10^{-8}$	+1.000	Significant

**Table 5:** Sensitivity analysis of MFCC parameters on fNIRS classification performance.

Frame Length (Samples)	Overlap (%)	MFCC (*N*)	Accuracy (%)
64	25	8	93.8
		10	93.9
		12	93.6
	50	8	94.3
		10	94.6
		12	94.5
	75	8	93.4
		10	93.7
		12	93.3
128	25	8	94.6
		10	94.8
		12	94.7
	50	8	94.8
		10	95.0
		12	94.7
	75	8	94.8
		10	94.0
		12	93.6
256	25	8	85.6
		10	86.7
		12	83.9
	50	8	86.9
		10	87.3
		12	85.2
	75	8	83.8
		10	84.2
		12	83.4

**Table 6:** Neural decoding accuracy (%) obtained using MFCCs combined with each of the twenty three-descriptor statistical subsets, averaged over 29 subjects under the same leave-one-subject-out protocol as Table 2. Values are reported as mean ± standard deviation across subjects.

Feature Combination	Accuracy (%)
MFCCs + (SM, SP, SD)	91.9±2.3
MFCCs + (SM, SP, KR)	92.1±2.1
MFCCs + (SM, SP, SK)	90.2±2.7
MFCCs + (SM, SP, VR)	90.5±2.6
MFCCs + (SD, SP, SK)	91.7±2.4
MFCCs + (SP, KR, VR)	89.6±3.0
MFCCs + (SP, SD, VR)	91.5±2.2
MFCCs + (VR, SD, KR)	91.0±2.5
MFCCs + (VR, SD, SM)	89.8±2.9
MFCCs + (VR, SM, SK)	90.2±2.8
MFCCs + (VR, SM, KR)	89.8±2.7
MFCCs + (KR, SP, SD)	88.5±3.1
MFCCs + (KR, SM, SK)	88.6±3.0
MFCCs + (SD, SM, SK)	88.3±3.2
MFCCs + (SD, SM, KR)	90.0±2.6
MFCCs + (SD, SK, VR)	88.6±3.0
MFCCs + (SD, SK, KR)	88.9±2.9
MFCCs + (SK, VR, KR)	87.9±3.2
MFCCs + (SK, SP, KR)	88.4±3.1
MFCCs + (SK, VR, SP)	89.0±2.9

**Table 7:** Leave-one-MFCC-coefficient-out analysis with temporal features.

Feature Set	Classification Accuracy (%)
Temporal + MFCC w/o $m_{1}$	94.3±0.4
Temporal + MFCC w/o $m_{2}$	95.1±0.3
Temporal + MFCC w/o $m_{3}$	95.0±0.5
Temporal + MFCC w/o $m_{4}$	95.4±0.2
Temporal + MFCC w/o $m_{5}$	94.9±0.4
Temporal + MFCC w/o $m_{6}$	94.7±0.3
Temporal + MFCC w/o $m_{7}$	95.2±0.5
Temporal + MFCC w/o $m_{8}$	94.1±0.4
Temporal + MFCC w/o $m_{9}$	94.5±0.3
Temporal + MFCC w/o $m_{10}$	94.2±0.5

**Table 8:** Comparative neural decoding performance (%) across HbO, HbR, and combined HbO+HbR chromophore representations under subject-independent LOSO cross-validation across 29 participants.

Chromophore	Feature Representation	Acc (%)	Prec (%)	Rec (%)	F1 (%)
HbR only	Temporal	85.2	88.1	84.0	86.0
	Cepstral (MFCC)	91.2	90.4	92.0	91.2
	Multi-domain (Concat.)	91.4	90.6	92.1	91.3
	Multi-domain (AMFF)	91.8	91.0	92.5	91.7
HbO only (main)	Temporal	90.7	93.1	89.2	91.1
	Cepstral (MFCC)	95.0	93.9	95.8	94.9
	Multi-domain (Concat.)	95.2	94.2	95.4	94.9
	Multi-domain (AMFF)	95.6	94.6	95.9	95.3
HbO + HbR	Temporal	91.5	93.8	90.4	92.1
	Cepstral (MFCC)	95.5	94.5	96.2	95.3
	Multi-domain (Concat.)	95.7	94.8	96.0	95.4
	Multi-domain (AMFF)	96.1	95.2	96.4	95.8

## Data Availability

Publicly available fNIRS data were analyzed in this study, available through the open-access repository provided by Shin et al. at http://doc.ml.tu-berlin.de/hBCI (accessed on 28 August 2025) (https://doi.org/10.1109/TNSRE.2016.2628057).
